# Supplementary material for: A silylene-stabilized ditin(0) complex and its conversion to methylditin cation and distannavinylidene
Source: Nat Commun. 2023 Nov 17;14:7474. doi: 10.1038/s41467-023-42953-5 (PMC10656547; doi:10.1038/s41467-023-42953-5)
Supplement: Supplementary file 1 — Supplementary Information [file 41467_2023_42953_MOESM1_ESM.pdf]

Supplementary Information for

A Silylene-Stabilized Ditin(0) Complex and Its Conversion to Methylditin Cation and Distannavinylidene

Shaozhi Du,[1] Fanshu Cao,[1] Xi Chen,[1] Hua Rong,[1] Haibin Song,[1] and Zhenbo Mo[1]*

[1]State Key Laboratory and Institute of Elemento-Organic Chemistry, Frontiers Science Center for New Organic Matter, College of Chemistry, Nankai University, Tianjin 300071, China

E-mail: zhenbo.mo@nankai.edu.cn

Table of Contents

1.Supplementary Methods 2

General Considerations: 2

Preparation of Compound **1**: 3

Preparation of Compound **2**: 6

Preparation of Compound **3**: 10

Preparation of Compound **4**: 14

Preparation of Compound **5**: 19

Reaction of **2** with $DippN_3$: 23

Reaction of LSi(NHI) (L= $PhC(N^tBu)_2$) with $DippN_3$: 26

Reaction of **2** with imQ: 29

Reaction of LSi(NHI) (L= $PhC(NtBu)_2$) with imQ: 32

Preparation of IPr→$SnBr_2$: 35

2. Supplementary Discussion 38

X-Ray Crystallography Data: 38

Computational Studies: 45

3. Supplementary References 54

1. Supplementary Methods

General Considerations

All reactions were performed under a dry and oxygen-free dinitrogen atmosphere using standard Schlenk line or under a dinitrogen atmosphere in a Vigor glovebox. The argon or dinitrogen in the glovebox was constantly circulated through a copper/molecular sieves catalyst unit. The oxygen and moisture concentrations in the glovebox atmosphere were monitored by an O_2/H_2O Combi-Analyzer (Vigor LG2400/750TS-F) to ensure both were always below 1 ppm. Samples for NMR spectroscopic measurements were prepared in the glovebox by use of J. Young valve NMR tubes. C_6D_6 and THF-d_8 was obtained from CIL and dried by fresh Na chips in the glovebox. ^{1}H, ^{13}C, ^{29}Si and ^{119}Sn NMR spectrum were recorded on a Bruker 400 MHz spectrometer at ambient temperature. Anhydrous THF, hexane, pentane, Et_2O and toluene were purified by use of Vigor Solvent Purification System, and dried over fresh Na chips in the glovebox. Fluorobenzene was stirred over CaH_2, trap-to-trap condensed and degassed by freeze-pump-thaw cycles. All commercially available chemicals were employed as received if not stated otherwise. IPr[1], LSi(NHI) (L= PhC(N^{t}Bu)$_2$)[2] and {(^{Mes}Nacnac)Mg}$_2$[3] was prepared according to literature procedures and was stored in the glovebox.

Synthetic Details

Preparation of 1:

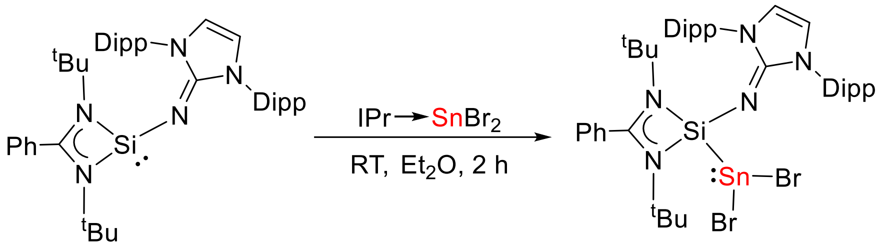

A Et_2O solution (5 mL) of LSi(NHI) (L= PhC(N^{t}Bu)$_2$) (662 mg, 1.0 mmol) was added to a stirred suspension of IPr→$SnBr_2$ (667 mg, 1.0 mmol) in Et_2O (5.0 mL) dropwise at room temperature. After stirring for 2 h, a yellowish slurry was obtained. The yellowish precipitate was isolated by filtration in 90% yield. Single crystals suitable for X-ray diffraction studies were obtained by vapor diffusion of *n*-hexane into the THF solution at room temperature.

^{1}H NMR (400 MHz, THF-d$_8$): δ 7.97 (d, J = 7.2 Hz, 1H, Ar*H*), 7.47-7.41 (m, 2H, Ar*H*), 7.39-7.35 (m, 4H, Ar*H*), 7.30-7.28 (m, 3H, Ar*H*), 7.17 (d, J = 7.6 Hz, 1H, Ar*H*), 6.73 (s, 2H, NC*H*), 3.14-3.04 (m, 4H, C*H*(CH$_3$)$_2$), 1.43 (d, J = 7.2 Hz, 12H, CH(C*H*$_3$)$_2$), 1.22 (d, J = 6.8 Hz, 12H, CH(C*H*$_3$)$_2$), 0.74 (s, 18H, C(C*H*$_3$)$_3$).

^{13}C{^{1}H} NMR (101 MHz, THF-d_8, 298 K): δ 173.4 (s, N*C*N), 148.1 (s, Ar*C*), 144.1 (s, Ar*C*), 135.2 (s, Ar*C*), 133.5 (s, Ar*C*), 132.1 (s, Ar*C*), 131.3 (s, Ar*C*), 130.6 (s, Ar*C*), 129.3 (s, Ar*C*), 128.8 (s, Ar*C*), 128.4 (s, Ar*C*), 125.4 (s, Ar*C*), 117.3 (s, N*C*H), 54.24 (s, *C*(CH$_3$)$_3$), 31.52 (s, *C*H(CH$_3$)$_2$), 29.66 (s, *C*H$_3$), 24.52 (s, *C*H$_3$). one peak for *C*H$_3$ are overlapped with the solvent residual signal of THF-d_8.

^{29}Si{^{1}H} NMR (79 MHz, THF-d_8, 298 K): δ -21.19 (s).

^{119}Sn{1H} NMR (149 MHz, THF-d_8, 298 K): δ 181.6 ($^1J_{Si, Sn}$ = 1245 Hz).

Anal. Calcd for $C_{42}H_{59}Br_2N_5SiSn$: C, 53.63; H, 6.32; N, 7.45. Found: C, 53.27; H, 6.43; N, 7.63.

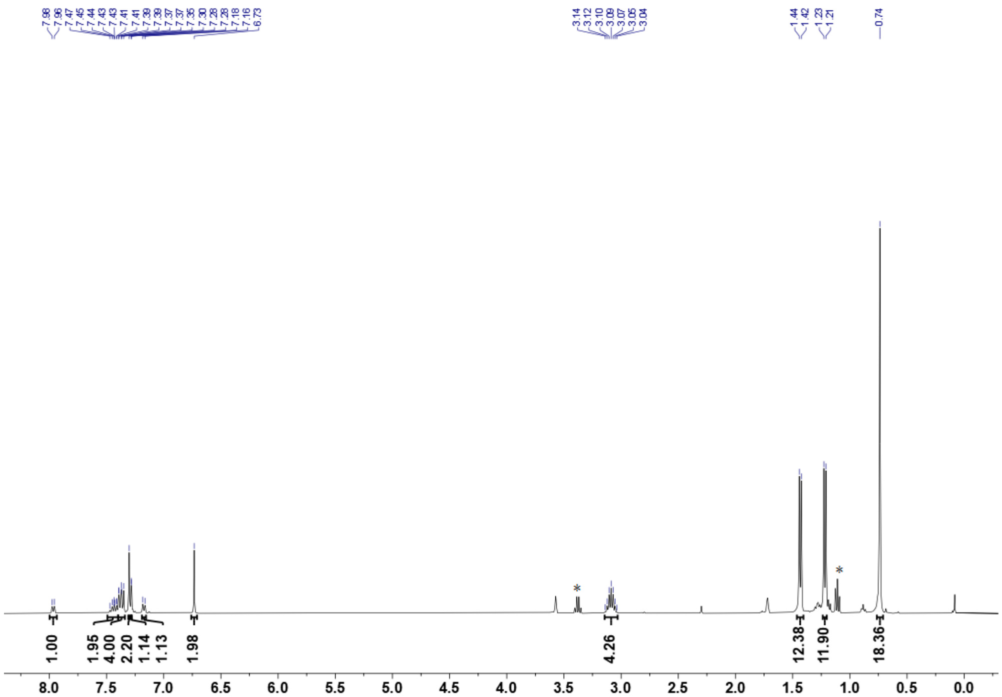

Supplementary Fig. 1. ^{1}H NMR spectrum of **1** in THF-d_8 at 298 K. Peaks with star are from Et_2O.

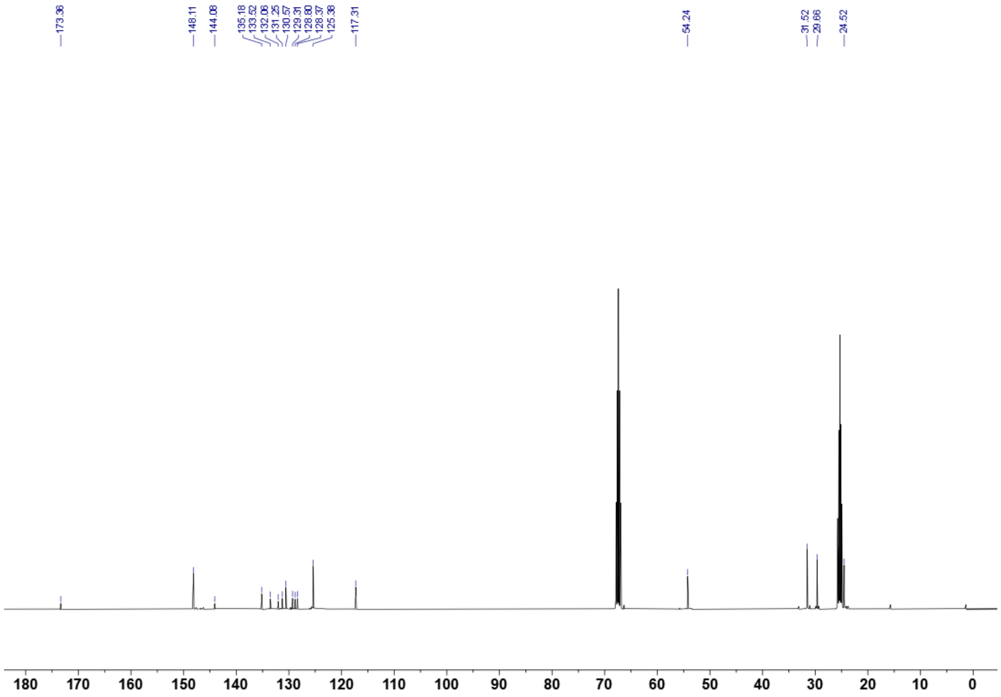

Supplementary Fig. 2. ^{13}C NMR spectrum of **1** in THF-d_8 at 298 K.

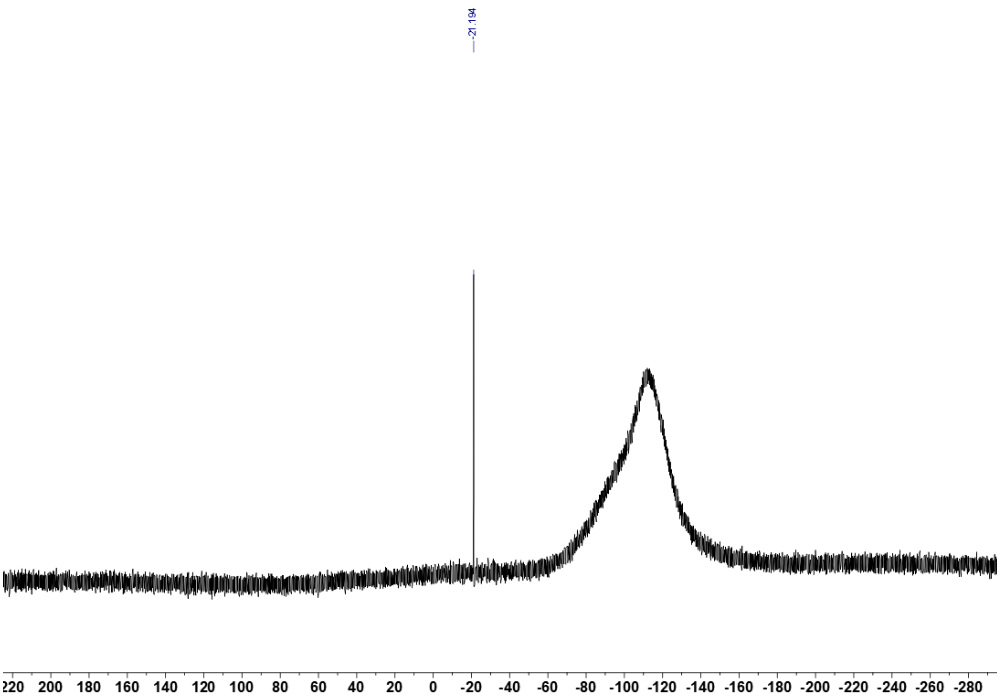

Supplementary Fig. 3. ^{29}Si NMR spectrum of **1** in THF-d_8 at 298 K.

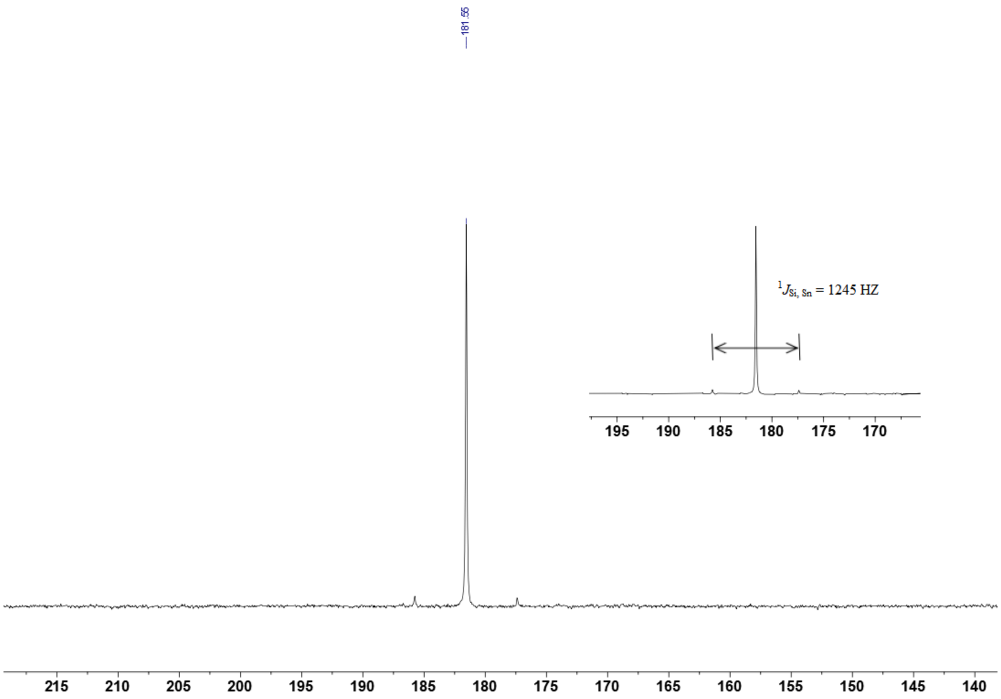

Supplementary Fig. 4. ^{119}Sn NMR spectrum of **1** in THF-d_8 at 298 K.

Preparation of 2:

Method A

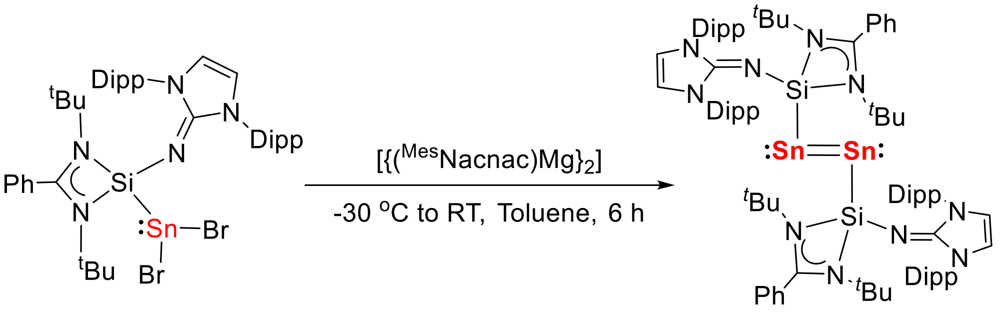

A toluene solution (10 mL) of {(*Mes*Nacnac)Mg}$_2$ (715.6 mg, 1.0 mmol) was added to a stirred suspension of 2 (940.6 mg, 1.0 mmol) in toluene (5.0 mL) dropwise at -30 ºC. The mixture was allowed to warm to room temperature and stirred for 4 h. Then the solvent was removed under vacuum and the resulting solids were extracted with 30 mL THF. After filtration and removal of the solvent, the resulted brown solid was washed with 60 mL Et_2O to yield dark green powder of **2** in 60% yield. Single crystals suitable for X-ray diffraction studies were grown by cooling the THF solution at -30 ºC.

Method B

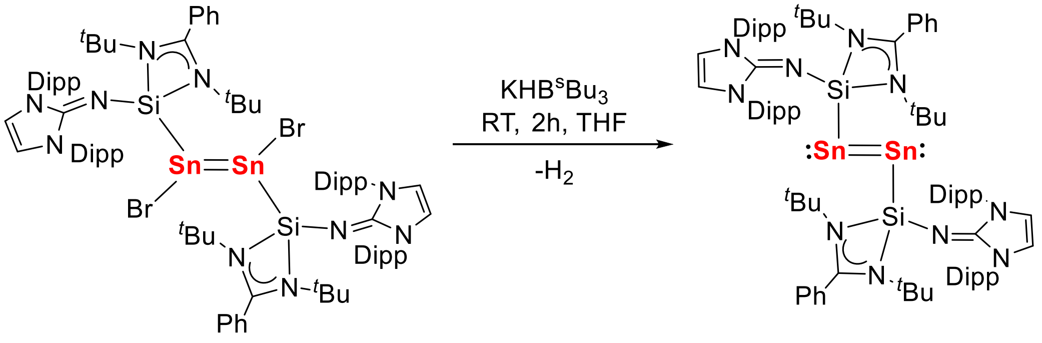

A THF solution (1.0 mL) of KHB^{s}Bu$_3$ (221.3 mg, 1.0 mmol) was added to the **3** (861 mg, 0.5 mmol) in THF (5.0 mL) dropwise at room temperature over a period of 2 minutes. The mixture was stirred for another 5 minutes. After filtration and removal of the solvent, the resulting brown

solid was washed with 5 mL Et_2O. Then the resulting solid was dissolved in a mixture of THF (10 mL) and *n*-hexane (5 mL). The suspension was filtered and the filtrate was stored at -30 ºC for 2 days to yield the green powder of **2** in 40% yield.

^{1}H NMR (400 MHz, THF-d_8): δ 8.54 (d, J = 6.8 Hz, 2H, Ar*H*), 7.39-7.29 (m, 7H, Ar*H*), 7.27-7.22 (m, 7H, Ar*H*), 7.16-7.15 (m, 6H, Ar*H*), 6.43 (s, 4H, NC*H*), 3.33-3.24 (m, 8H, C*H*(CH_3)$_2$), 1.34 (d, J = 6.4 Hz, 24H, CH(CH_3)$_2$), 1.16 (d, J = 6.8 Hz, 24H, CH(CH_3)$_2$), 0.72 (s, 36H, C(CH_3)$_3$).The solubility in THF-d_8 is not good enough.

^{13}C{^{1}H} NMR (101 MHz, THF-d_8, 298 K): δ 170.1 (s, N*C*N), 149.1 (s, Ar*C*), 145.7 (s, Ar*C*), 137.6 (s, Ar*C*), 136.6 (s, Ar*C*), 135.0 (s, Ar*C*), 130.1 (s, Ar*C*), 129.8 (s, Ar*C*), 129.7(s, Ar*C*), 127.8 (s, Ar*C*), 127.7 (s, Ar*C*), 124.8 (s, Ar*C*), 116.6 (s, N*C*H), 54.16 (s, *C*(CH_3)$_3$), 32.03 (s, *C*H(CH_3)$_2$), 29.54 (s, *C*H_3), 24.76 (s, *C*H_3). one peak for *C*H_3 are overlapped with the solvent residual signal of THF-d_8.

^{29}Si{^{1}H} NMR (79 MHz, THF-d_8, 298 K): δ -5.89 (s).

^{119}Sn{^{1}H} NMR (149 MHz, THF-d_8, 298 K): no signal was observed in the range from -3000 to 3000 ppm, most likely due to the anisotropy of the shift tensor.

Absorption spectrum (THF): λmax (ε) = 587 (8713), 459(5715) and 264(78513) nm.

Anal. Calcd for $C_{84}H_{118}N_{10}Si_2Sn_2$: C, 64.61; H, 7.62; N, 8.97. Found: C, 64.81; H, 7.75; N, 8.95.

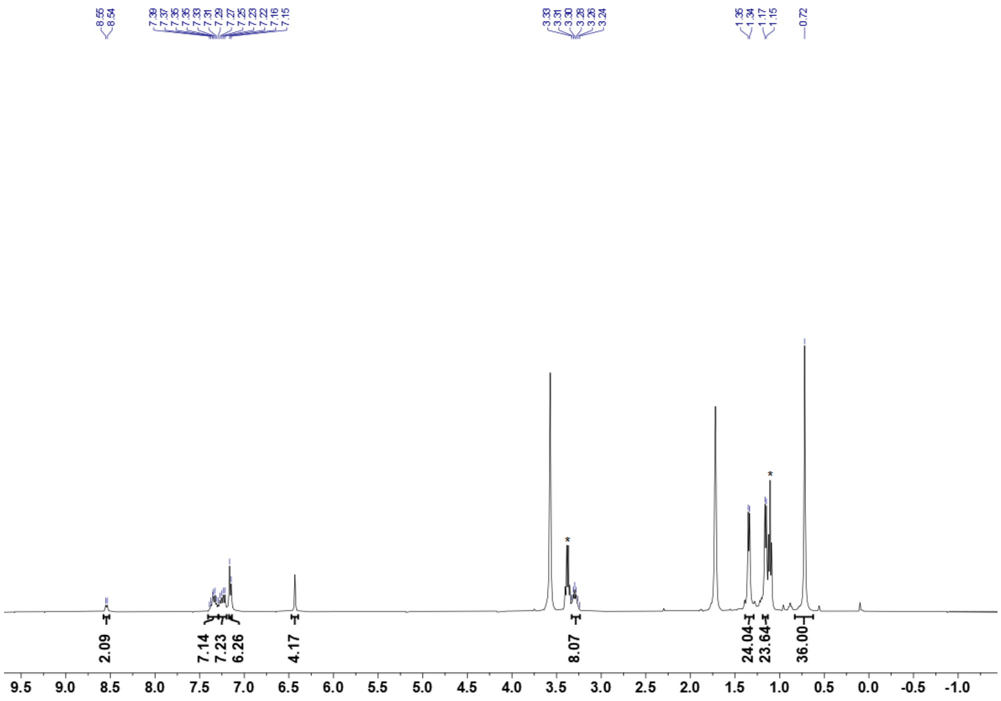

Supplementary Fig. 5. ^{1}H NMR spectrum of **2** in THF-d_8 at 298 K. Peaks with star are from Et_2O.

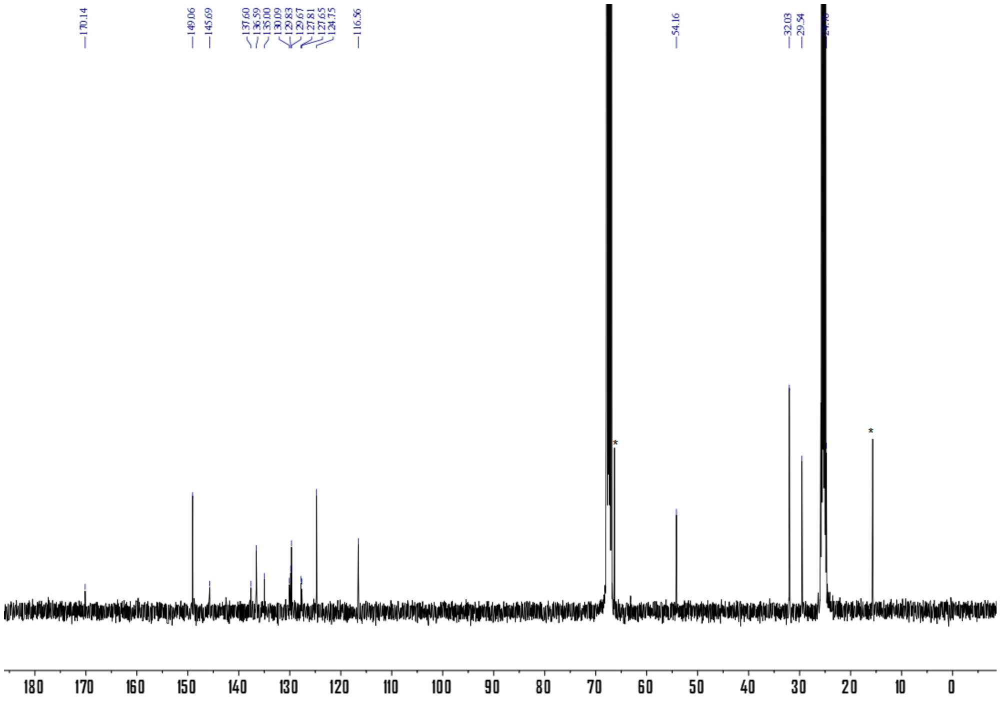

Supplementary Fig. 6. ^{13}C NMR spectrum of **2** in THF-d_8 at 298 K. Peaks with star are from Et_2O.

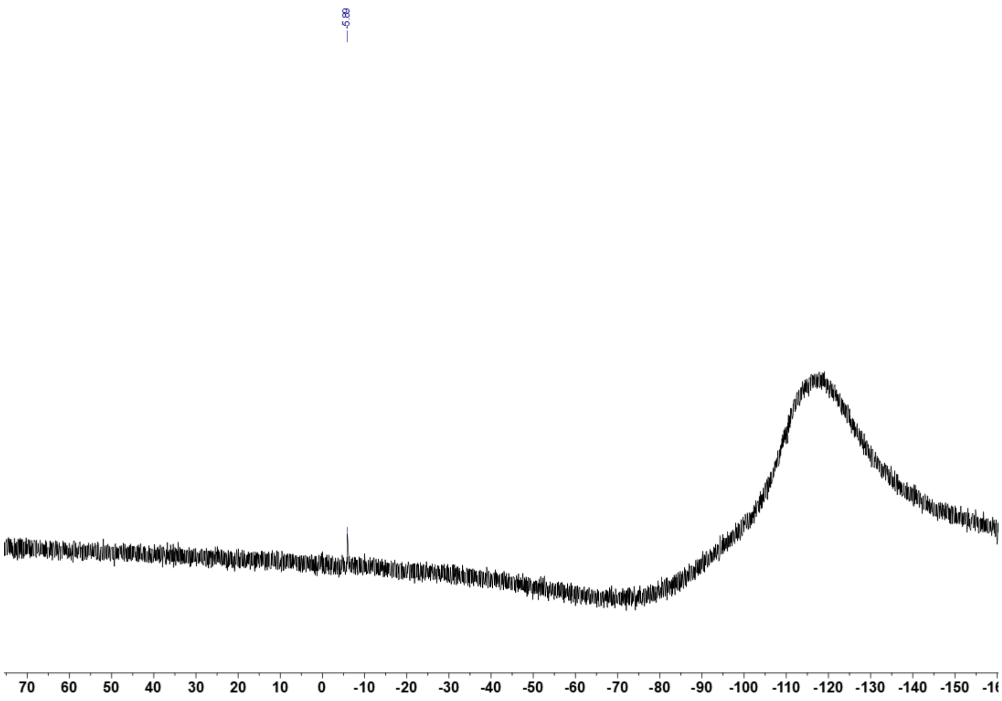

Supplementary Fig. 7. ^{29}Si NMR spectrum of **2** in THF-d_8 at 298 K.

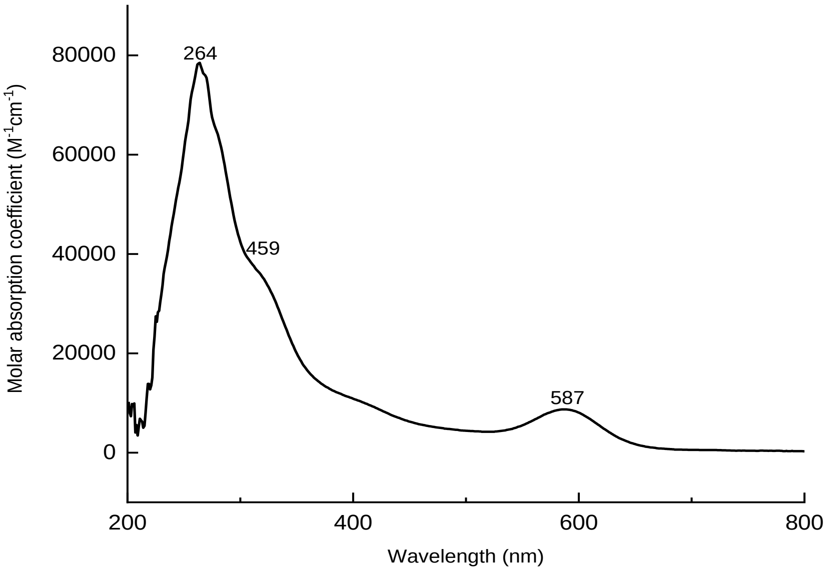

Supplementary Fig. 8. UV/Vis spectrum of compound **2** at room temperature.

Preparation of 3:

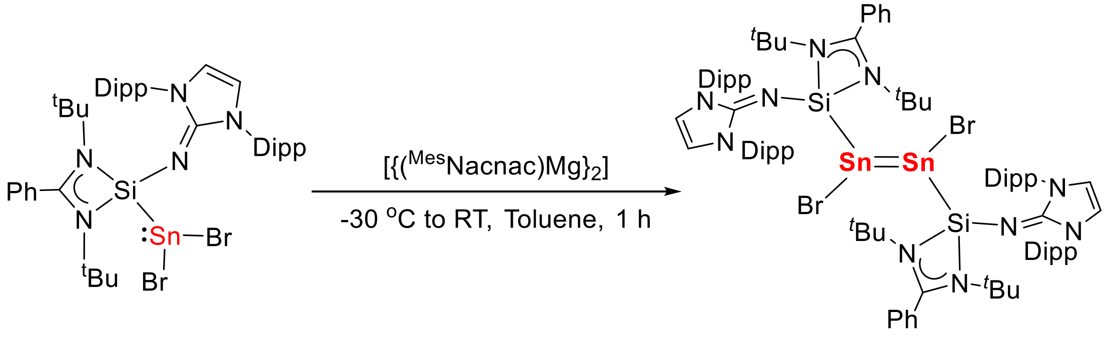

A toluene solution (10 mL) of $\{(^{Mes}Nacnac)Mg\}_2$ (357.8 mg, 0.5 mmol) was added to a stirred suspension of **2** (940.6 mg, 1.0 mmol) in toluene (5.0 mL) dropwise at -30 ºC. The mixture was allowed to warm to room temperature and stirred for 0.5 h. After removal of the solvent, the resulted solid was washed with 30 mL Et_2O to yield green powder of **3** in 90 % yield. Single crystals suitable for X-ray diffraction studies were obtained by vapor diffusion of *n*-hexane into the benzene solution at room temperature.

^1H NMR (400 MHz, THF-d_8): δ 8.15 (d, J = 7.2 Hz, 2H, Ar*H*), 7.39-7.28 (m, 12H, Ar*H*), 7.25-7.23 (m, 6H, Ar*H*), 7.14 (d, J = 8.0 Hz, 2H, Ar*H*), 6.84 (s, 4H, NC*H*), 3.31-3.21 (m, 8H, C*H*$(CH_3)_2$), 1.47 (d, J = 6.8 Hz, 24H, $CH(CH_3)_2$), 1.18 (d, J = 6.8 Hz, 24H, $CH(CH_3)_2$), 0.79 (s, 36H, $C(CH_3)_3$).

$^{13}C\{^1H\}$ NMR (101 MHz, THF-d_8, 298 K): δ 171.2 (s, N*C*N), 148.8 (s, Ar*C*), 144.1 (s, Ar*C*), 136.2 (s, Ar*C*), 133.2 (s, Ar*C*), 130.2 (s, Ar*C*), 129.71 (s, Ar*C*), 129.69 (s, Ar*C*), 128.9 (s, Ar*C*), 128.1 (s, Ar*C*), 127.5 (s, Ar*C*), 124.9 (s, Ar*C*), 116.8 (s, N*C*H), 53.70 (s, $C(CH_3)_3$), 31.74 (s, $CH(CH_3)_2$), 29.31 (s, CH_3), 25.18 (s, CH_3). one peak for CH_3 is overlapped with the solvent residual signal of THF-d_8.

$^{29}Si\{^1H\}$ NMR (79 MHz, THF-d_8, 298 K): δ -23.29 (s).

$^{119}Sn\{^1H\}$ NMR (149 MHz, THF-d_8, 298 K): δ 143.6 ($^1J_{Si,\,Sn}$ = 1208 Hz).

Absorption spectrum (THF): λmax (ε) = 342 (24201) and 263 (72193) nm.

Anal. Calcd for $C_{84}H_{118}Br_2N_{10}Si_2Sn_2$: C, 58.61; H, 6.91; N, 8.14. Found: C, 58.45; H, 6.83; N, 8.34.

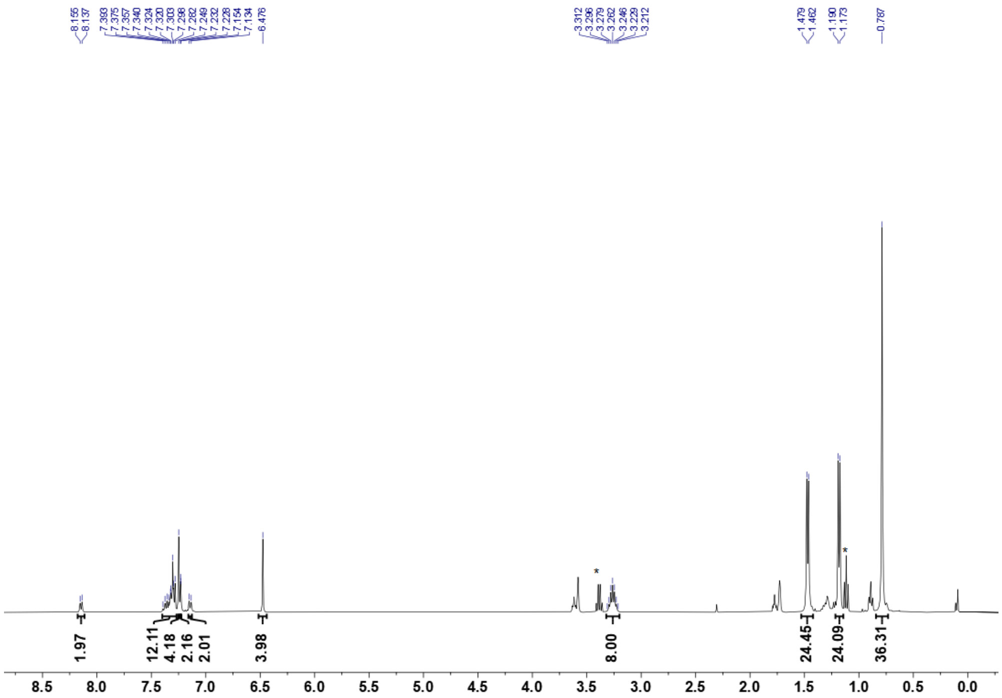

Supplementary Fig. 9. ^{1}H NMR spectrum of **3** in THF-d_8 at 298 K. Peaks with star are from Et_2O.

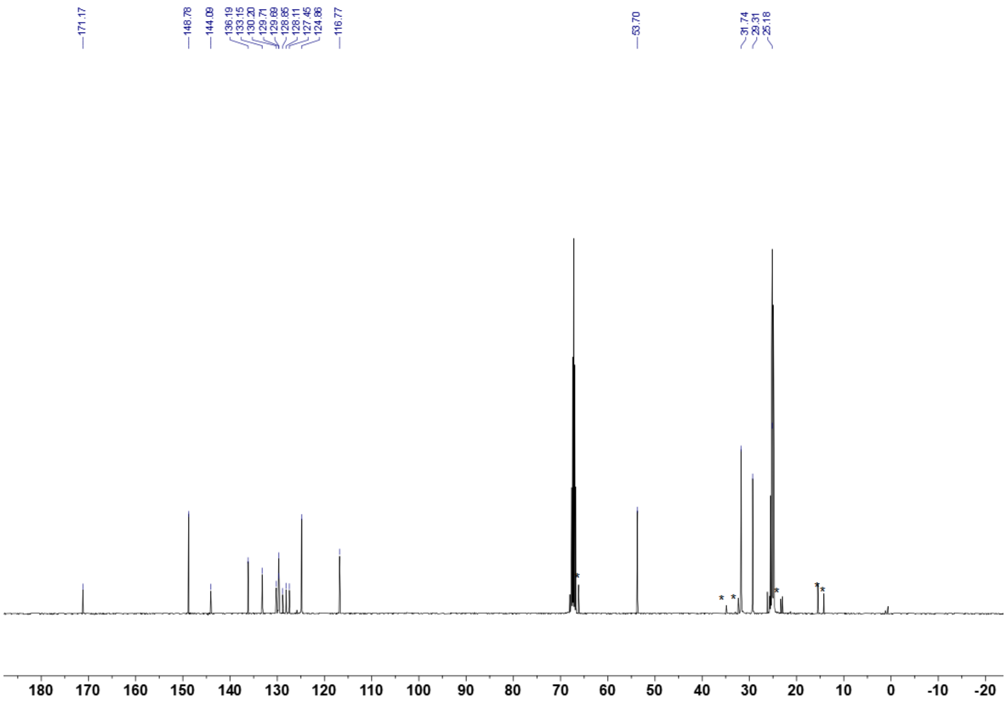

Supplementary Fig. 10. ^{13}C NMR spectrum of **3** in THF-d_8 at 298 K. Peaks with star are from hexane, pantane and Et_2O.

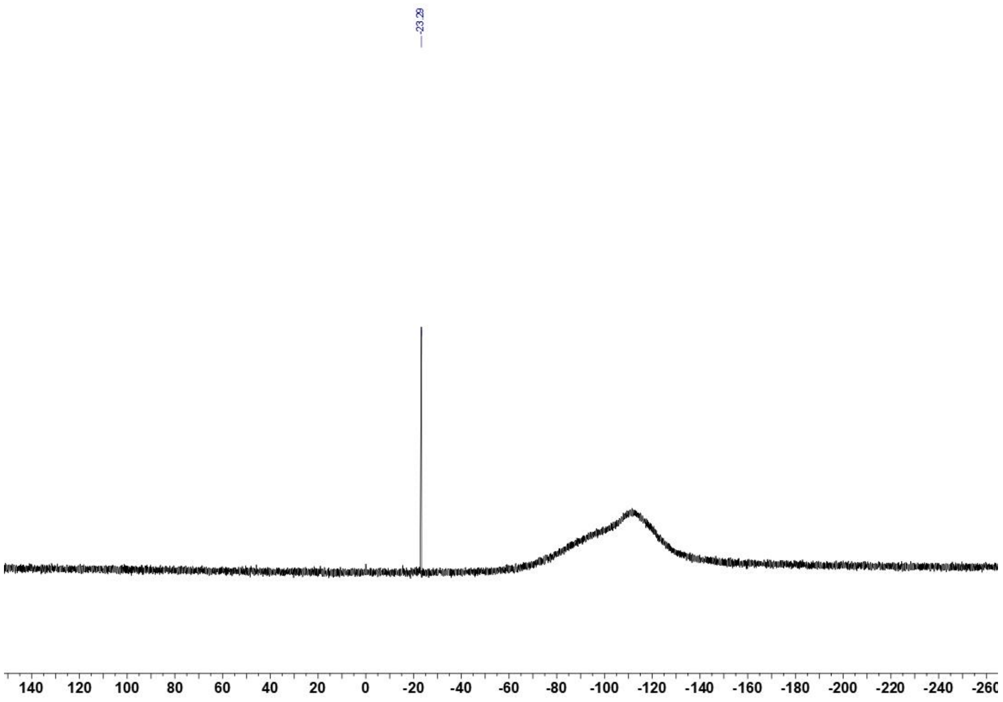

Supplementary Fig. 11. ^{29}Si NMR spectrum of **3** in THF-d_8 at 298 K.

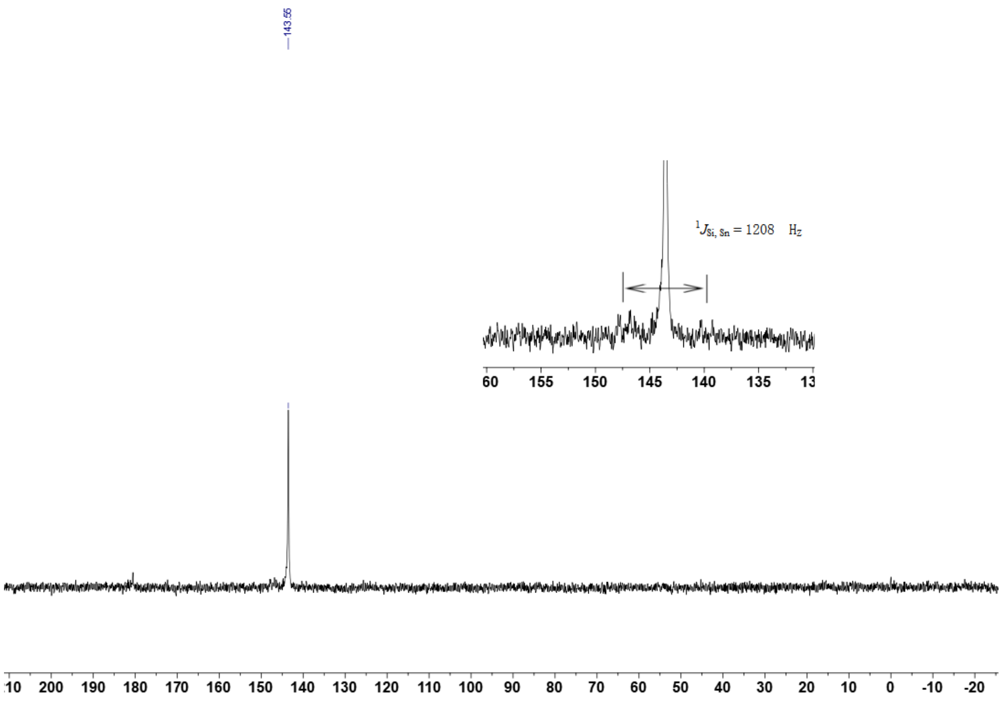

Supplementary Fig. 12. ^{119}Sn NMR spectrum of **3** in THF-d_8 at 298 K.

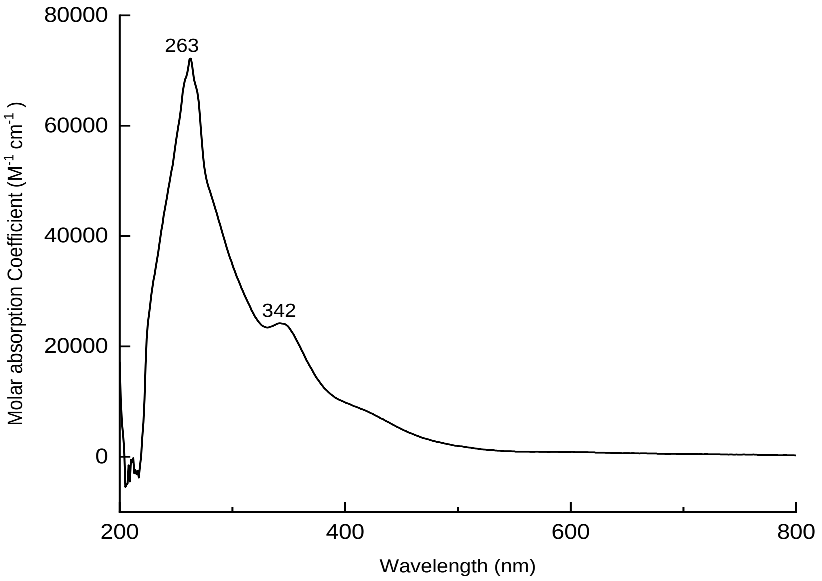

Supplementary Fig. 13. UV/Vis spectrum of compound **3** at room temperature.

Preparation of 4:

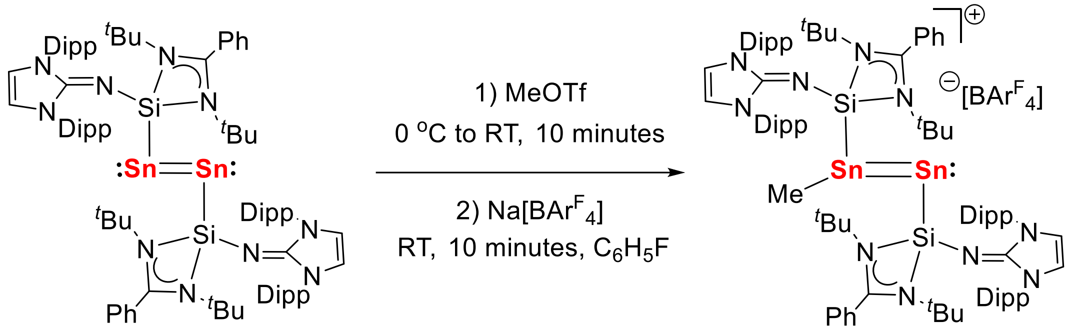

A toluene solution (10 mL) of MeOTf (83.5 mg, 0.5 mmol) was added to the solution of **2** (780.8 mg, 0.5 mmol) in fluorobenzene (10 mL) dropwise at 0 ºC over a period of 10 minutes. The mixture was allowed to warm to room temperature. Then a fluorobenzene solution (10 mL) of Na[B(Ar^F)$_4$](Ar^F = C_6H_3-3,5-(CF_3)$_2$) (443 mg, 0.5 mmol) was added dropwise over a period of 2 minutes. The solution was stirred at room temperature for 10 minutes and the solvent was removed under reduced pressure. Then the resulting solids were extracted with 30 mL toluene. After filtration and removal of the solvent, the resulted purple solid was washed with 30 mL hexane to yield purple powder of **4** in 90 % yield. Single crystals suitable for X-ray diffraction studies were obtained by layering *n*-pentane on a fluorobenzene solution of **4** at room temperature.

^{1}H NMR (400 MHz, C_6D_6, 298 K): δ 8.44 (s, 8H, B(Ar^F)$_4$-Ar*H*), δ 7.71-7.69 (m, 6H, Ar*H*), 7.14-7.06 (m, 14H, Ar*H*), 7.03-6.96 (m, 4H, Ar*H*), 6.00 (s, 4H, NC*H*), 3.27-3.02 (m, 8H, C*H*(CH_3)$_2$), 1.52 (s, 3H, Sn*C*H$_3$),1.40 (d, J = 6.4 Hz, 24H, CH(C*H*$_3$)$_2$), 1.13 (d, J = 6.8 Hz, 24H, CH(C*H*$_3$)$_2$), 0.74 (s, 36H, C(C*H*$_3$)$_3$). one peak for Ar*H* is overlapped with B(Ar^F)$_4$-Ar*H*. Several peaks for Ar*H* are overlapped with the solvent residual signal of C_6D_6.

^{13}C{^{1}H} NMR (101 MHz, C_6D_6, 298 K): δ 174.0 (s, N*C*N), 162.9 (q, $J_{C\text{-}B}$ = 50 Hz, B(Ar^F)$_4$-Ar-*C*), 147.5 (s, Ar*C*), 144.8 (s, Ar*C*), 135.5 (s, B(Ar^F)$_4$-Ar-*C*), 134.1 (s, Ar*C*), 131.0 (s, Ar*C*), 130.6

(s, Ar*C*), 130.4 (s, Ar*C*), 129.9 (m, B(Ar^F)$_4$-Ar-*C*), 129.3 (s, Ar*C*), 129.2 (s, Ar*C*), 125.7 (s, Ar*C*), 125.3 (q, J_{C-F} = 274 Hz, B(Ar^F)$_4$-*C*F_3),124.8 (s, Ar*C*),118.1 (m, B(Ar^F)$_4$-Ar-*C*), 116.4 (s, N*C*H), 54.75 (*C*(CH_3)$_3$), 31.21 (*C*H(CH_3)$_2$), 28.93 (*C*H_3), 25.14(*C*H_3), 23.85 (*C*H_3). The resonances for Sn*C*H_3 can not be found because of topomerization.

^{19}F NMR (377 MHz, C_6D_6, 298 K): δ -62.06 (s).

^{11}B NMR (128 MHz, C_6D_6, 298 K): δ -11.09 (s).

^{29}Si{^{1}H} NMR (79 MHz, C_6D_6, 298 K): no signal was observed due to the topomerization.

^{119}Sn{^{1}H} NMR (149 MHz, C_6D_6, 298 K): no signal was observed due to the topomerization.

^{119}Sn{^{1}H} NMR (149 MHz, THF-d_8, 213 K): δ 633.4 (s, Me*Sn*Sn) and 297.9 (s, MeSn*Sn*).

Absorption spectrum (THF): λmax (ε) = 531 (8295) nm and 263 (90223) nm.

Anal. Calcd for $C_{117}H_{133}BF_{24}N_{10}Si_2Sn_2$: C, 57.60; H, 5.49; N, 5.74. Found: C, 57.45; H, 5.61; N, 5.57.

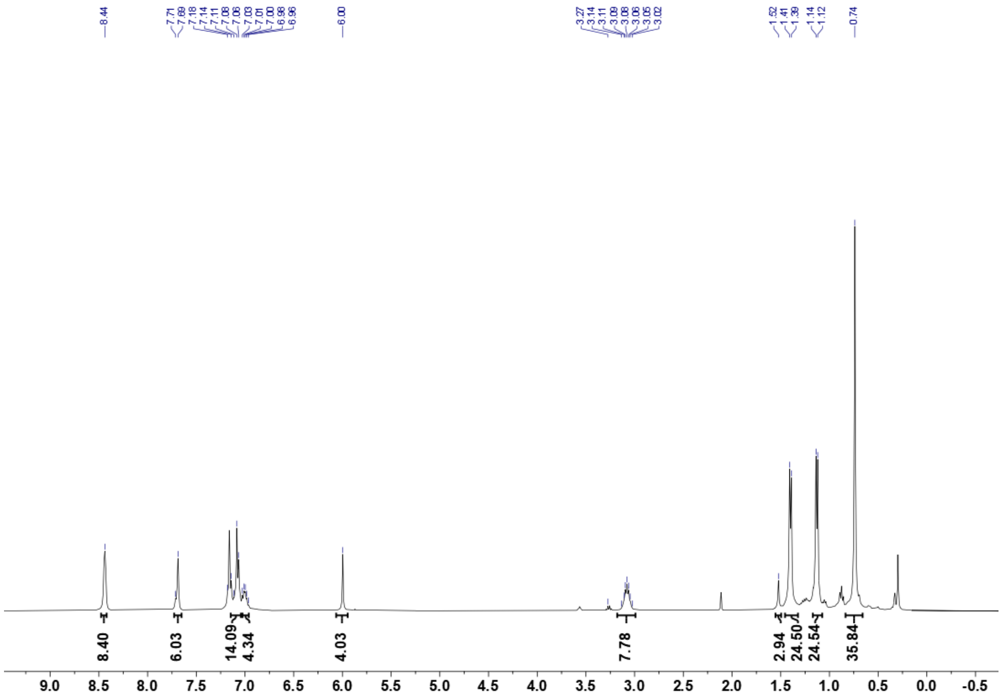

Supplementary Fig. 14. ^{1}H NMR spectrum of **4** in C_6D_6 at 298 K.

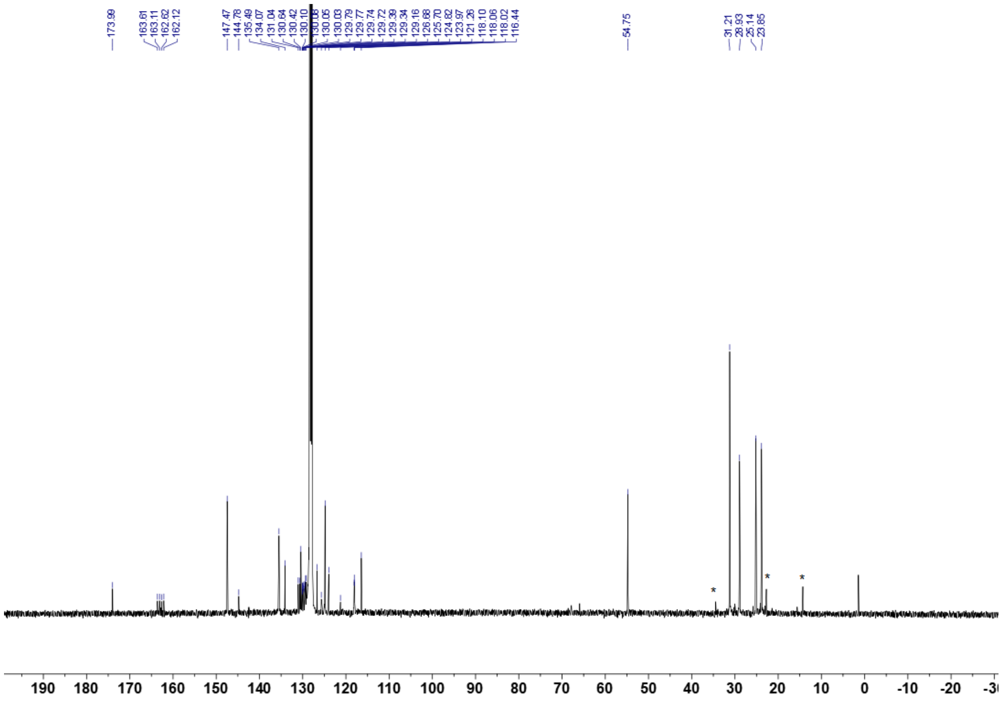

Supplementary Fig. 15. ^{13}C NMR spectrum of **4** in C_6D_6 at 298 K. Peaks with star are from pantane.

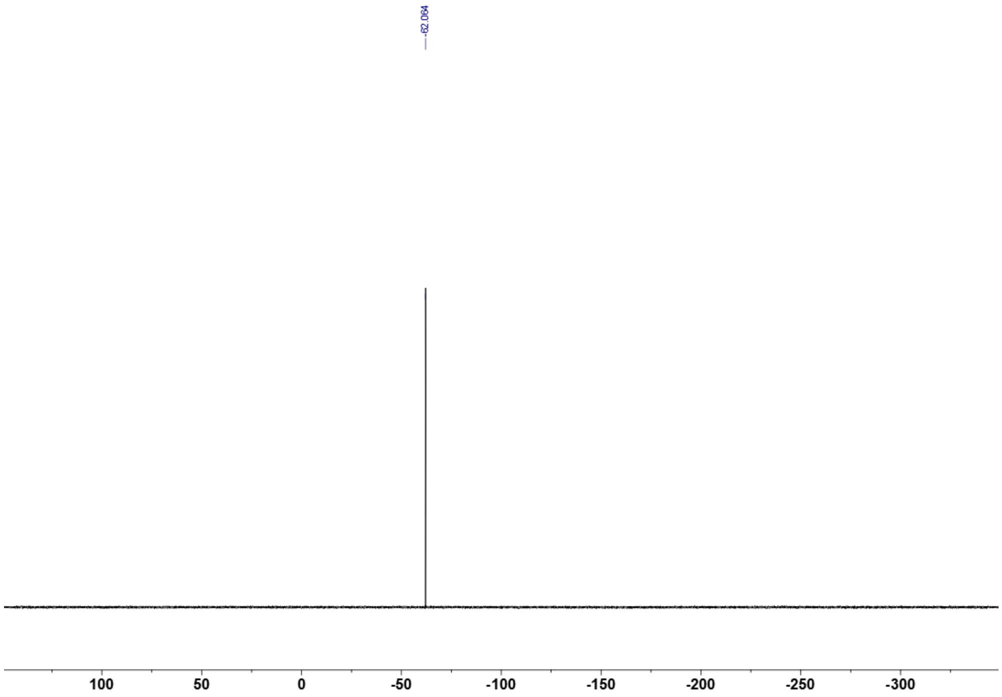

Supplementary Fig. 16. ^{19}F NMR spectrum of **4** in C_6D_6 at 298 K.

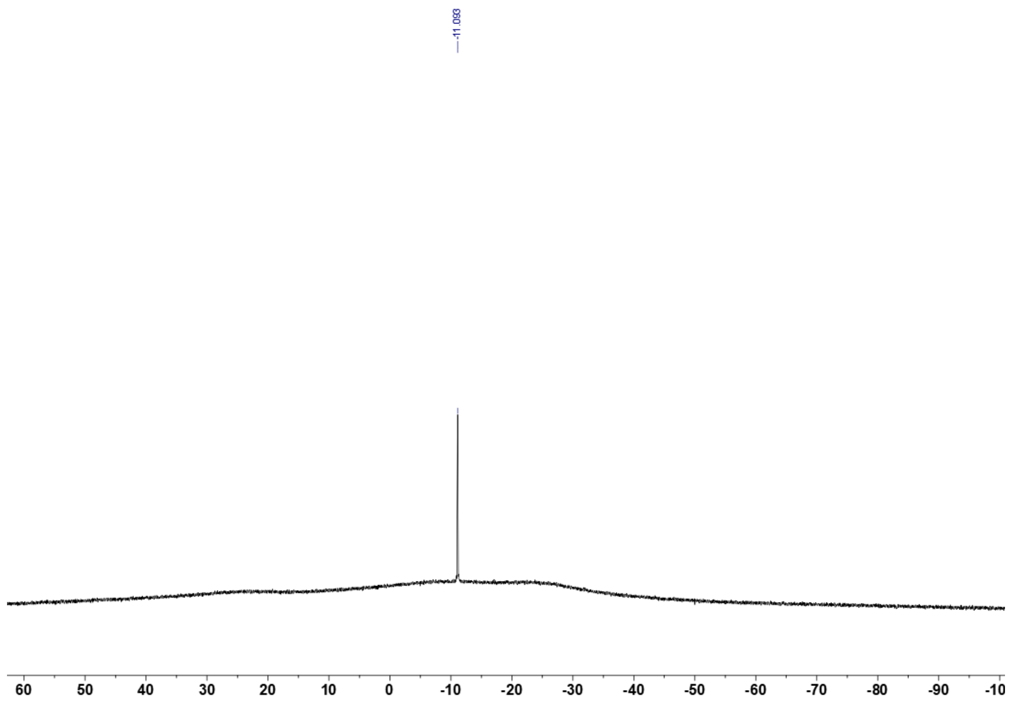

Supplementary Fig. 17. ^{11}B NMR spectrum of **4** in C_6D_6 at 298 K.

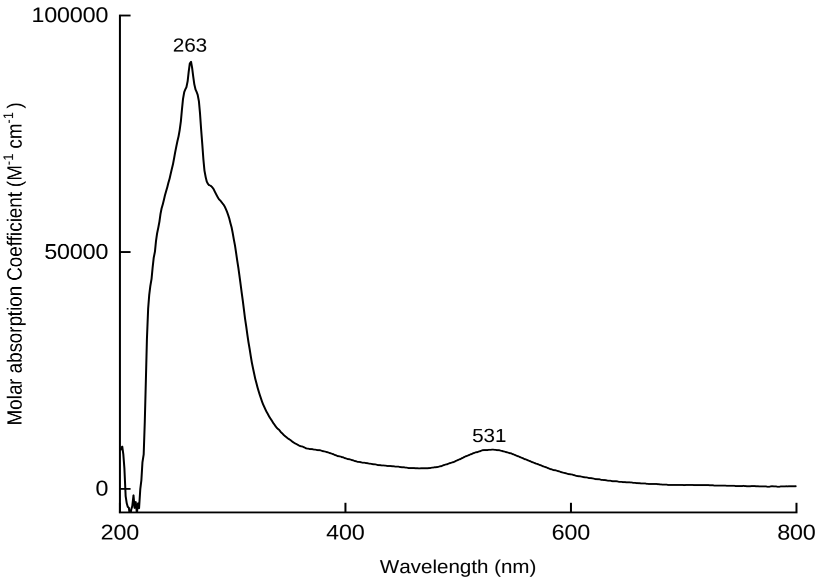

Supplementary Fig. 18. UV/Vis spectrum of compound **4** at room temperature.

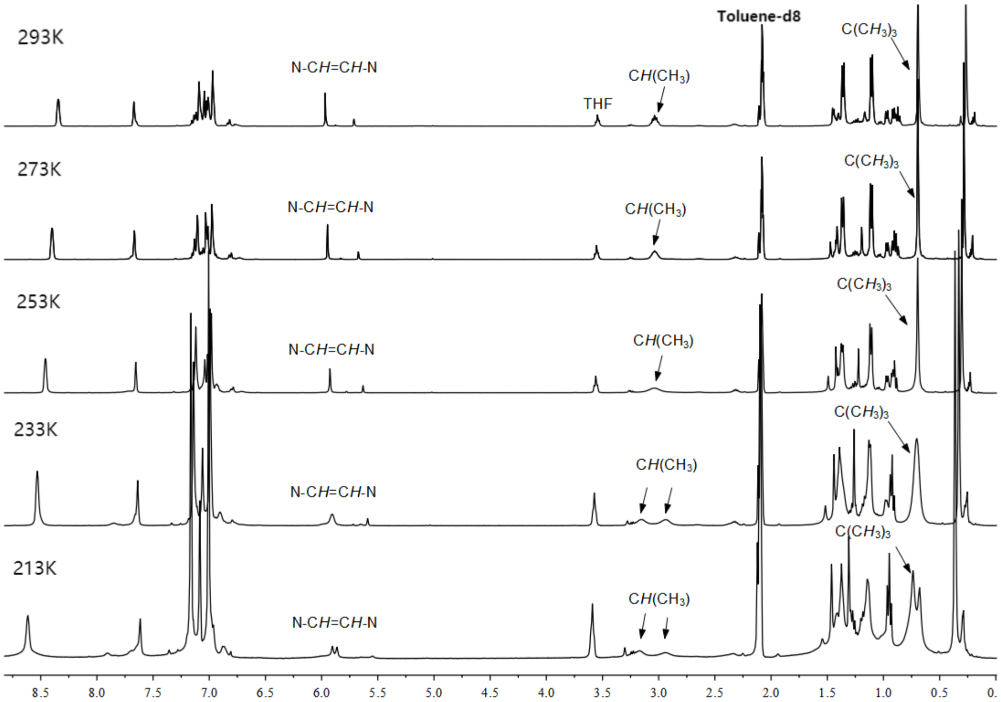

Supplementary Fig. 19. Variable temperature ^{1}H NMR spectra of compound **4** in toluene-d_8 in the temperature range 213-293 K.

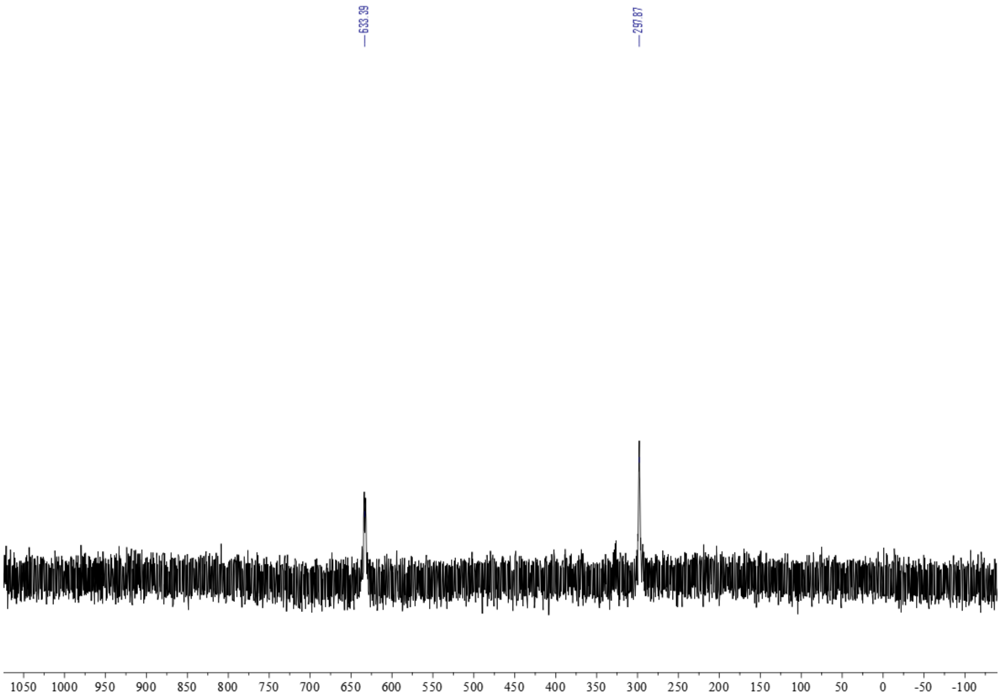

Supplementary Fig. 20. ^{119}Sn NMR spectrum of **4** in THF-d_8 at 213 K.

Preparation of 5:

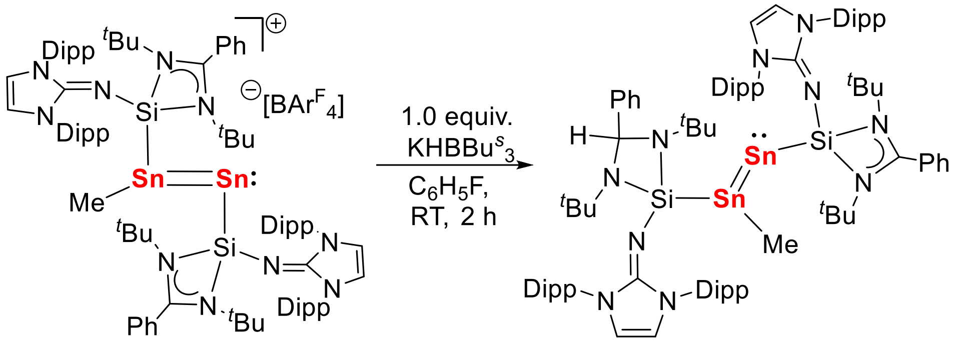

Method A: A THF solution (1mL) of KHB^{s}Bu$_3$ (0.5 mmol) was added to the solution of **4** (1088.2 mg, 0.5 mmol) in fluorobenzene (10 mL) dropwise. The solution was stirred at room temperature for 2 h and then the solvent was removed under reduced pressure. Then the resulting solids were extracted with a mixture of Et_2O (10 mL) and *n*-hexane (20 mL). After filtration and removal of the solvent, the resulted solid was dissolved in a mixture of Et_2O (10 mL) and *n*-hexane (5 mL). The suspension was filtered and the filtrate was stored at -30 ºC for 2 days to yield the purple powder of **5** in 30% yield.

Method B:

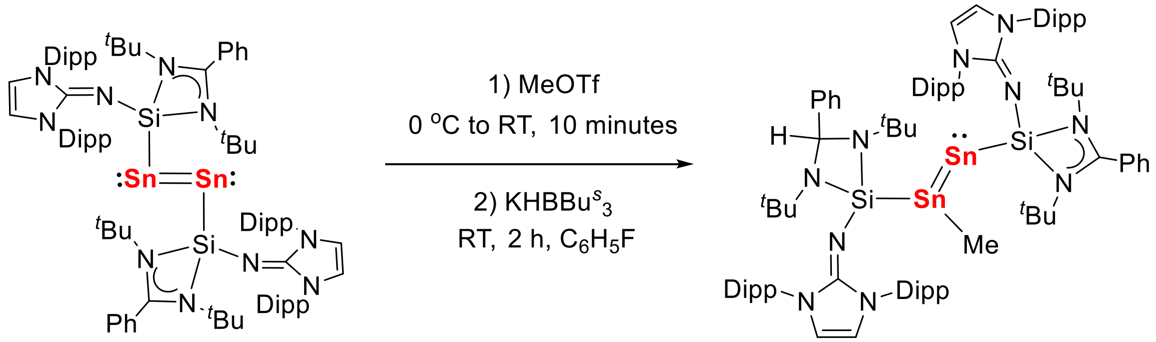

A toluene solution (10 mL) of MeOTf (83.5 mg, 0.5 mmol) was added to the solution of **2** (780.8 mg, 0.5 mmol) in fluorobenzene (10 mL) dropwise at 0 ºC over a period of 10 minutes. The mixture was allowed to warm to room temperature and a THF solution (1mL) of KHB^{s}Bu$_3$ (0.5 mmol) was added. The solution was stirred at room temperature for 2 h and then the solvent was

removed under reduced pressure. Then the resulting solids were extracted with 30 mL Et_2O. After filtration and removal of the solvent, the resulted solid was dissolved in a mixture of Et_2O (10 mL) and n-hexane (5 mL). The suspension was filtered and the filtrate was stored at -30 ºC for 2 days to yield the purple powder of **5** in 70% yield. Single crystals suitable for X-ray diffraction studies were obtained by evaporation of a Et_2O solution at room temperature.

^{1}H NMR (400 MHz, C_6D_6, 298 K): δ 8.12 (d, *J* = 7.6 Hz, 1H, Ar*H*), δ 7.95 (d, *J* = 6.8 Hz, 1H, Ar*H*), 7.42 (t, *J* = 6.8 Hz, 1H, Ar*H*), 7.31 (d, *J* = 6.8 Hz, 1H, Ar*H*), 7.21-7.19 (m, 2H, Ar*H*), 7.15-7.08 (m, 10H, Ar*H*), 6.96-7.92 (m, 2H, Ar*H*), 6.04 (s, 2H, NC*H*), 6.02 (s, 2H, NC*H*), 5.51 (s, 1H, NC*H*N), 3.57-3.48 (m, 4H, C*H*(CH_3)$_2$), 3.45-3.36 (m, 4H, C*H*(CH_3)$_2$), 1.65 (d, *J* = 6.8 Hz, 12H, CH(CH_3)$_2$), 1.25 (d, *J* = 6.8 Hz, 12H, CH(CH_3)$_2$), 1.16 (d, *J* = 6.8 Hz, 12H, CH(CH_3)$_2$), 0.98 (s, 18H, *t*Bu), 0.84 (s, 18H, C(CH_3)$_3$). Several peaks for Ar*H* are overlapped with the solvent residual signal of C_6D_6.one peak for SnCH_3 is overlapped with the CH(CH_3)$_2$.

^{13}C{^{1}H} NMR (101 MHz, C_6D_6, 298 K): δ 171.4 (s, N*C*N), 151.8 (s, Ar*C*), 148.4 (s, Ar*C*),148.3 (s, Ar*C*), 145.6 (s, Ar*C*), 135.6 (s, Ar*C*), 135.3 (s, Ar*C*), 132.7 (s, Ar*C*), 131.4 (s, Ar*C*), 130.0 (s, Ar*C*), 129.8 (s, Ar*C*), 129.5 (s, Ar*C*), 127.5 (s, Ar*C*), 127.1 (s, Ar*C*), 127.0 (s, Ar*C*), 126.8 (s, Ar*C*), 124.6 (s, Ar*C*), 124.3 (s, Ar*C*), 116.0 (s, N*C*H), 115.2 (s, N*C*H), 72.33 (N*C*HN), 54.35 (*C*(CH_3)$_3$), 51.05 (*C*(CH_3)$_3$), 31.57 (*C*H(CH_3)$_2$), 31.44 (*C*H(CH_3)$_2$), 28.97 (*C*H_3), 28.84 (*C*H_3), 25.71 (*C*H_3), 25.67 (*C*H_3), 24.57 (*C*H_3), 24.29 (*C*H_3), 13.23 (Sn*C*H_3). one peak for Ar*C* is overlapped with the solvent residual signal of C_6D_6.

^{29}Si{^{1}H} NMR (79 MHz, C_6D_6, 298 K): δ -28.83 (s, *Si*MeSnSnSi) and -7.40 (s, SiMeSnSn*Si*)

^{119}Sn{^{1}H} NMR (149 MHz, C_6D_6+THF, 298 K): δ 843.2 (s, Me*Sn*Sn) and 300.3 (s, MeSn*Sn*)

Absorption spectrum (THF): λmax (ε) = 396 (12115) nm, 533 (18078) nm, 264 (103043) nm and 224 (130650) nm.

Anal. Calcd for $C_{85}H_{122}N_{10}Si_2Sn_2$: C, 64.72; H, 7.80; N, 8.88. Found: C, 64.64; H, 7.66; N, 8.65.

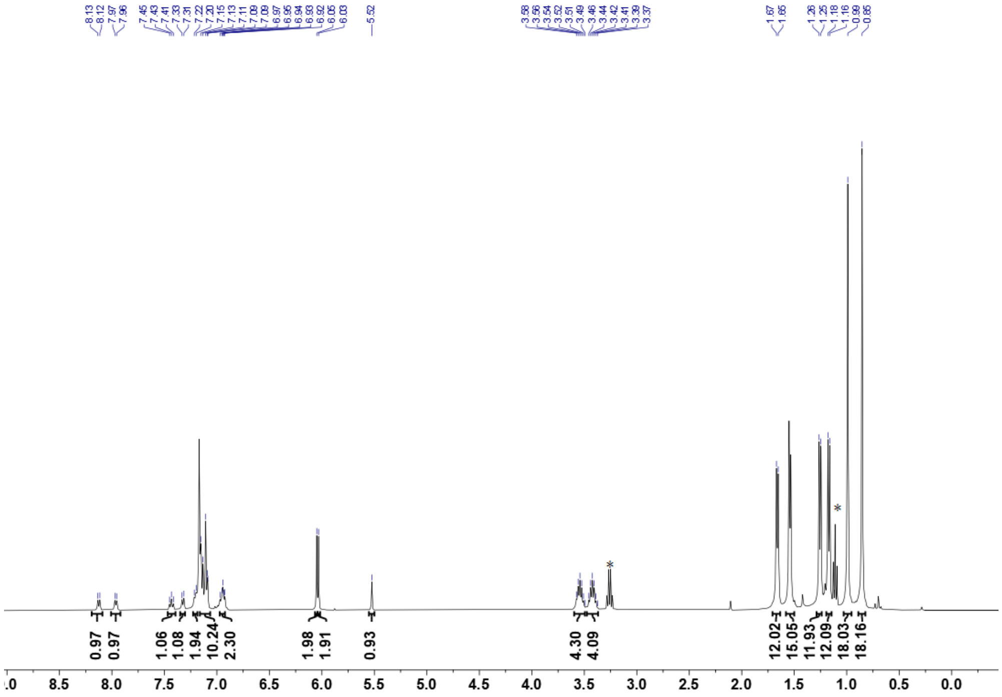

Supplementary Fig. 21. ^{1}H NMR spectrum of **5** in C_6D_6 at 298 K. Peaks with star are from Et_2O.

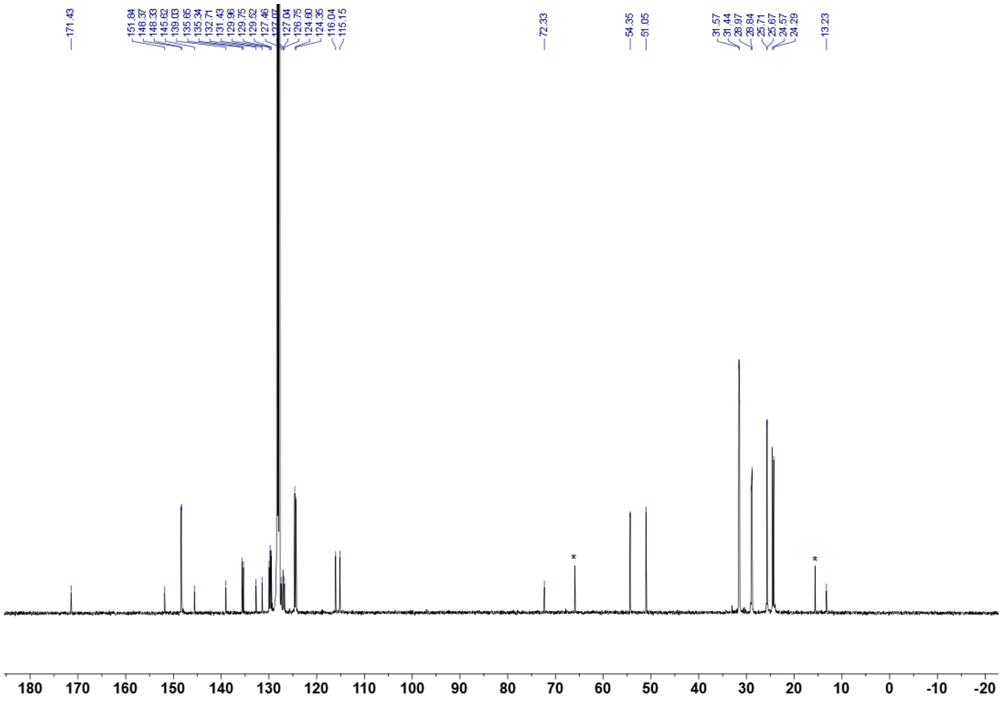

Supplementary Fig. 22. ^{13}C NMR spectrum of **5** in C_6D_6 at 298 K. Peaks with star are from Et_2O.

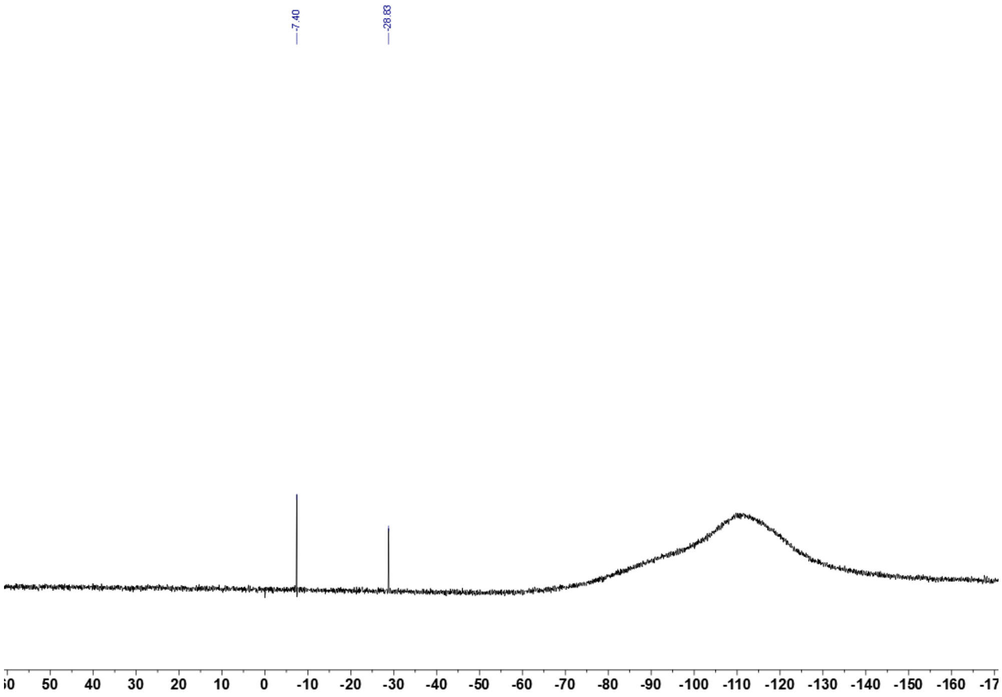

Supplementary Fig. 23. ^{29}Si NMR spectrum of **5** in C_6D_6 at 298 K.

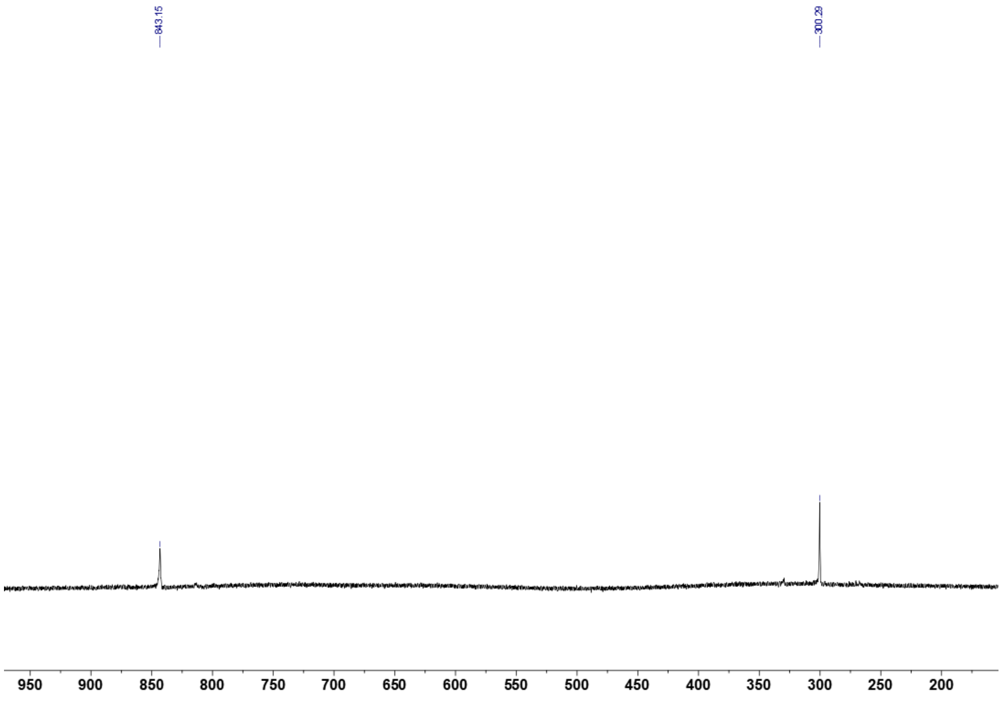

Supplementary Fig. 24. ^{119}Sn NMR spectrum of **5** in C_6D_6+THF at 298 K.

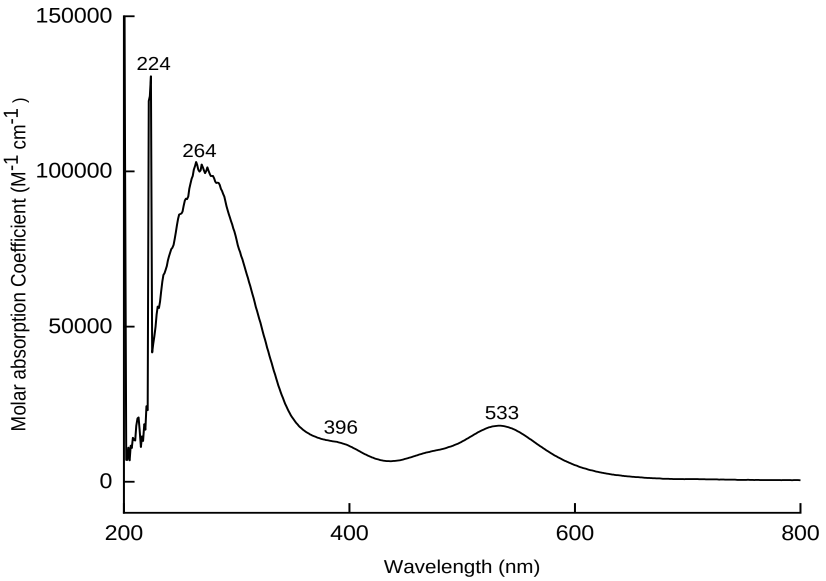

Supplementary Fig. 25. UV/Vis spectrum of compound **5** at room temperature.

Reaction of 2 with $DippN_3$:

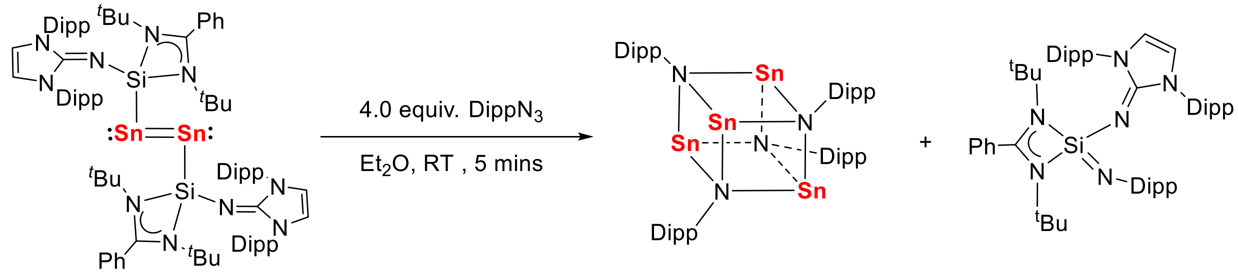

A Et_2O suspension (10 mL) of **2** (312 mg, 0.2 mmol) was added to the $DippN_3$ (163 mg, 0.8 mmol) in Et_2O (10 mL) over 2 min at room temperature leading to immediate gas evolution and a color change to yellow. The solvent was removed in vacuum and subsequent NMR analysis revealed clean formation of $(SnNDipp)_4$ and silaimine compound (from the reaction of LSi(NHI) (L= $PhC(N^tBu)_2$) with the $DippN_3$: See blow) with the ratio 1:4. Characteristic NMR data of $(SnNDipp)_4$: ^1H, ^{13}C, and ^{119}Sn NMR spectrum of $(SnNDipp)_4$ are in agreement with reported literature[4].

^{1}H NMR (400 MHz, C_6D_6, 298 K): 1.29 (d, J = 6.4 Hz, 48H, CH(CH_3)$_2$). Several peaks for CH(CH_3)$_2$ and ArH are partially overlapped with the peaks for the silaimine compound.

^{13}C{^{1}H} NMR (101 MHz, C_6D_6, 298 K): δ 148.1 (s, ArC), 142.0 (ArC), 125.4 (ArC), 121.3 (s, ArC), 30.59 (CH(CH_3)$_2$), 27.97 (CH(CH$_3$)$_2$).

^{119}Sn{^{1}H} NMR (149 MHz, C_6D_6, 298 K): δ 316.9

^{29}Si{^{1}H} NMR (79 MHz, C6D6, 298 K): δ -105.57

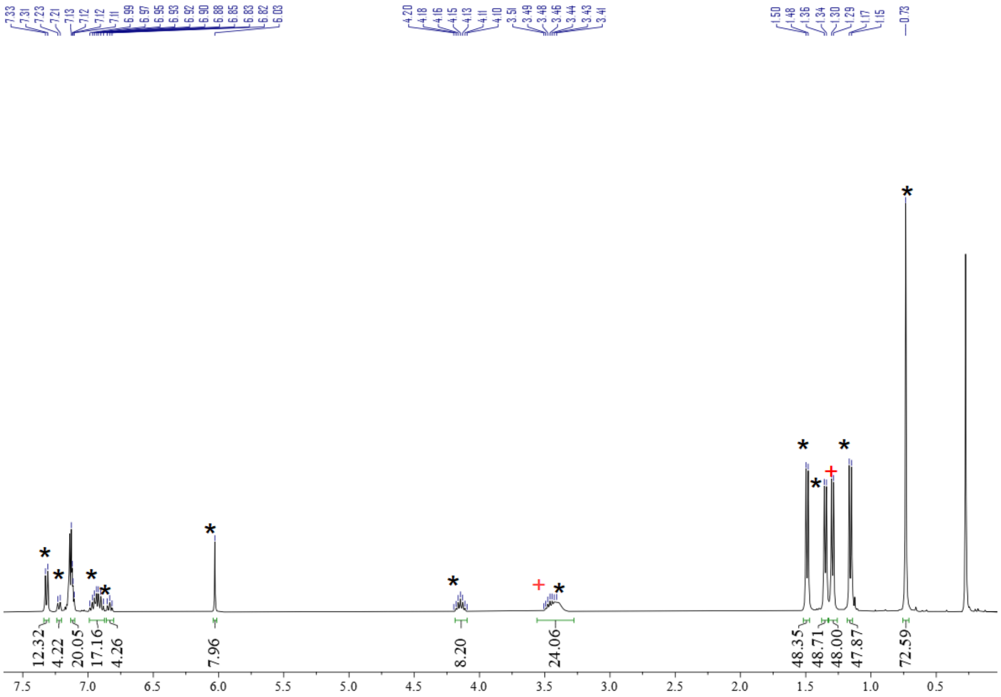

Supplementary Fig. 26. ^{1}H NMR spectrum after the reaction of **2** with DippN_3 in C_6D_6 at 298 K. Peaks with **+** are from (SnNDipp)$_4$. Peaks with ***** are from silaimine compound.

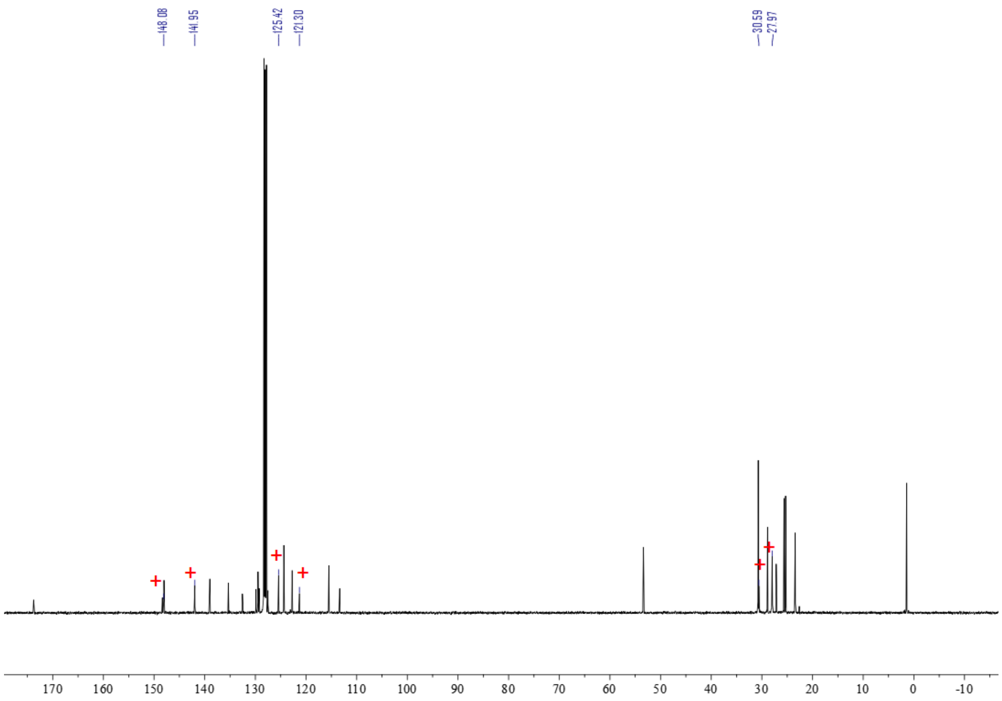

Supplementary Fig. 27. ^{13}C NMR spectrum after the reaction of **2** with $DippN_3$ in C_6D_6 at 298 K. Peaks with + are from $(SnNDipp)_4$.

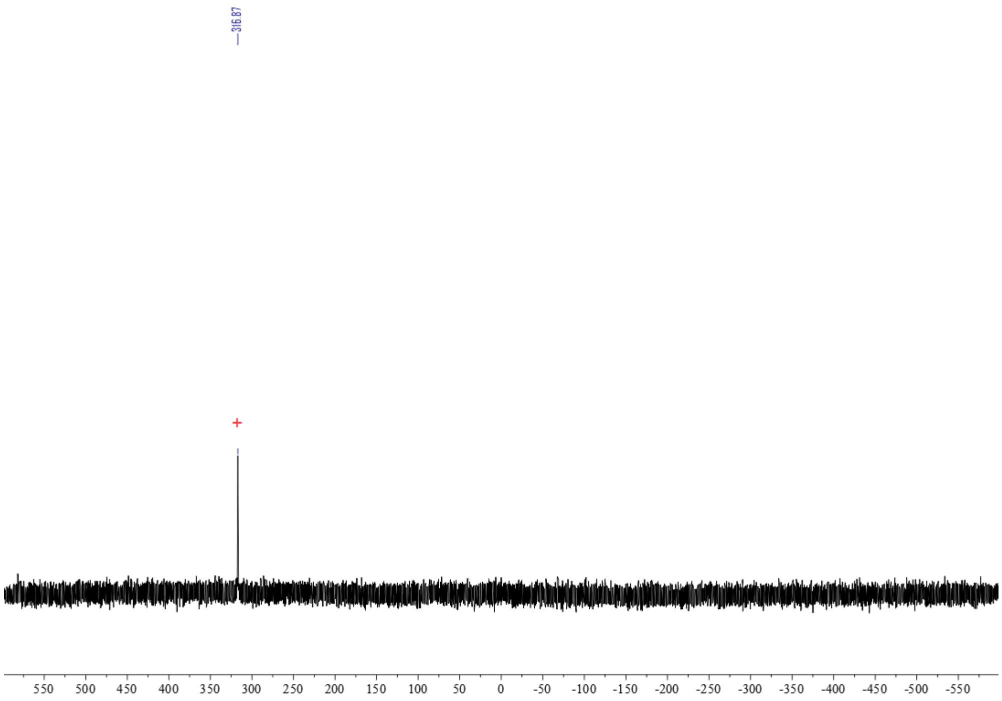

Supplementary Fig. 28. ^{119}Sn NMR spectrum after the reaction of **2** with $DippN_3$ in C_6D_6 at 298 K. Peaks with + are from $(SnNDipp)_4$.

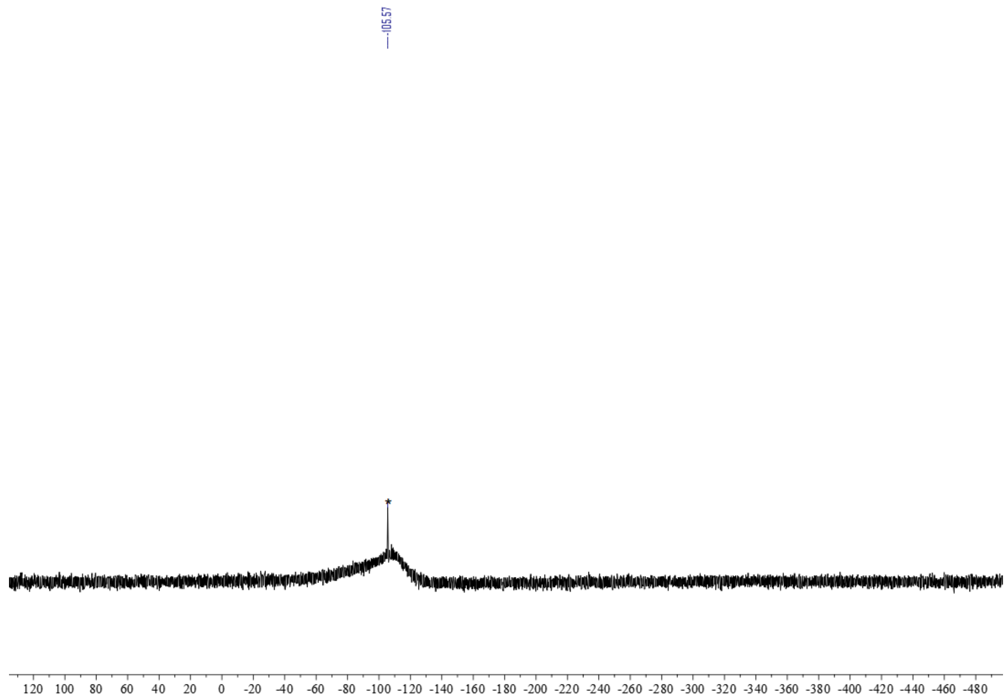

Supplementary Fig. 29. ^{29}Si NMR spectrum after the reaction of **2** with $DippN_3$ in C_6D_6 at 298 K.Peaks with ***** are from silaimine compound.

Reaction of LSi(NHI) (L= PhC(N^tBu)$_2$) with $DippN_3$:

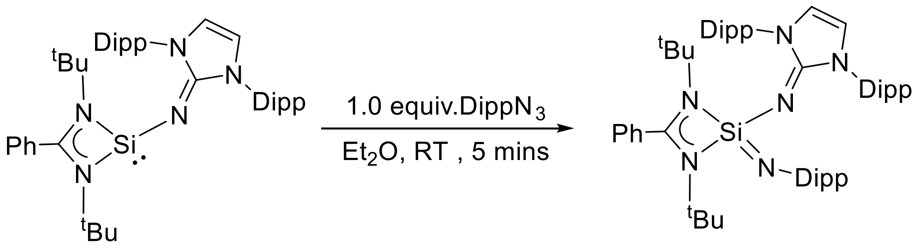

A Et_2O solution (1.0 mL) of $DippN_3$ (203.3 mg, 1.0 mmol) was added to the LSi(NHI) (L= PhC(N^tBu)$_2$) (662 mg, 1.0 mmol) in Et_2O (5.0 mL) dropwise at room temperature over a period of 2 minutes. After removal of the solvent, the resulting yellow solid was obtained in 98% yield.

^{1}H NMR (400 MHz, C_6D_6, 298 K): δ 7.34-7.31 (m, 3H, Ar*H*), 7.23 (d, *J* = 7.6 Hz, 1H, Ar*H*), 7.19-7.18 (m, 1H, Ar*H*), 7.15-7.12 (m, 5H, Ar*H*), 6.04 (s, 4H, NC*H*), 4.20-4.10 (m, 2H, C*H*(CH_3)$_2$), 3.42 (s, br, 4H, C*H*(CH_3)$_2$),1.49 (d, *J* = 6.8 Hz, 12H, CH(CH_3)$_2$), 1.36 (d, *J* = 6.8 Hz, 12H, CH(CH_3)$_2$), 1.17 (d, *J* = 6.8 Hz, 12H, CH(CH_3)$_2$), 0.74 (s, 18H, C(CH_3)$_3$).

$^{13}C\{^1H\}$ NMR (101 MHz, C_6D_6, 298 K): δ 173.80 (s, N*C*N), 148.38 (s, Ar*C*), 148.02 (s, Ar*C*), 141.98 (s, Ar*C*), 138.98 (s, Ar*C*), 135.32 (s, Ar*C*), 132.53 (s, Ar*C*), 129.90 (s, Ar*C*), 129.48 (s, Ar*C*), 129.23 (s, Ar*C*), 127.54 (s, Ar*C*), 124.37 (s, Ar*C*), 122.73 (s, Ar*C*), 115.48 (s, Ar*C*), 113.31 (s, Ar*C*), 53.39 (*C*(CH_3)$_3$), 30.72 (*C*H(CH_3)$_2$), 28.87 (*C*H(CH_3)$_2$), 27.17 (*C*H_3), 25.63 (*C*H_3), 25.32 (*C*H_3), 23.42 (*C*H_3). Two peaks for Ar*H* are overlapped with the solvent residual signal of C_6D_6.

$^{29}Si\{^1H\}$ NMR (79 MHz, C_6D_6, 298 K): δ -105.54.

Anal. Calcd for $C_{54}H_{76}N_6Si$: C, 77.46; H, 9.15; N, 10.04. Found: C, 76.91; H, 9.57; N, 10.55.

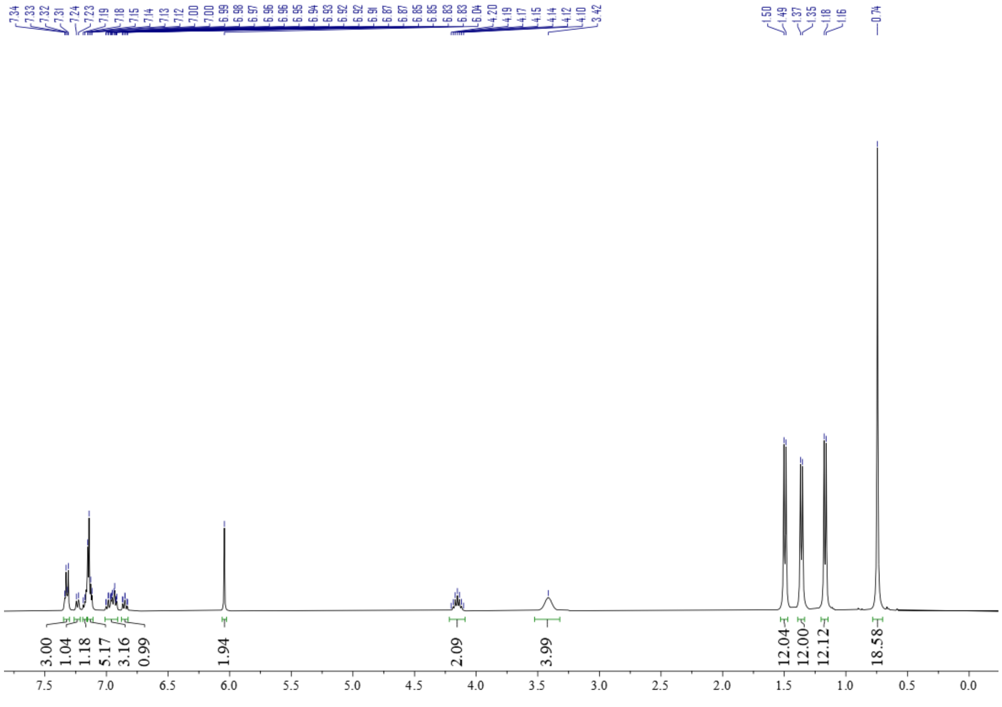

Supplementary Fig. 30. ^{1}H NMR spectrum after the reaction of LSi(NHI) with $DippN_3$ in C_6D_6 at 298 K.

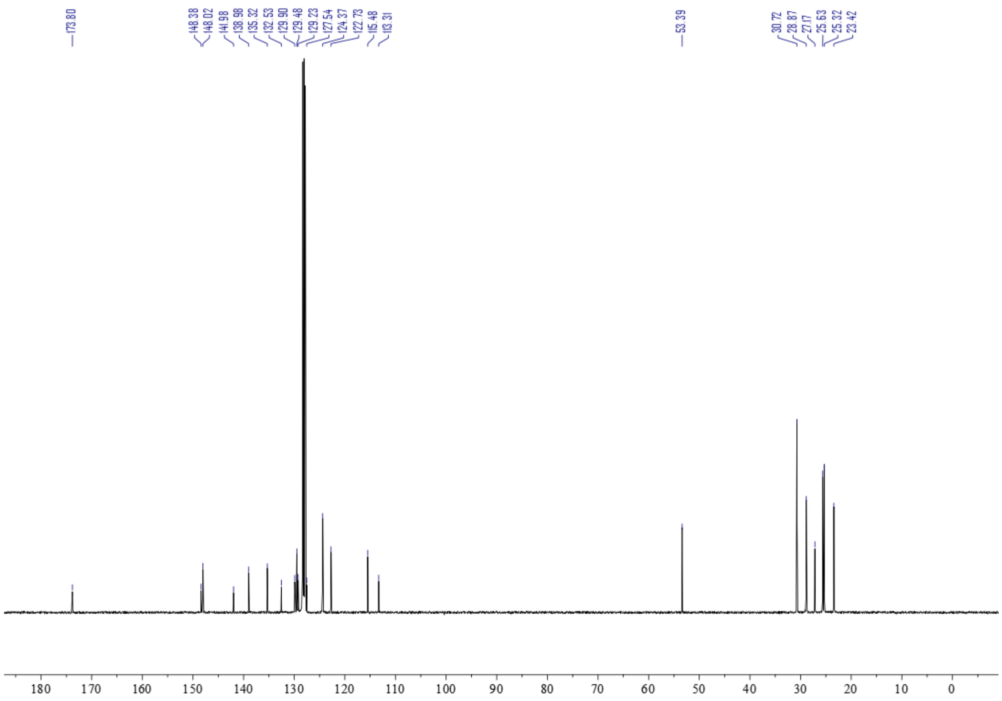

Supplementary Fig. 31. ^{13}C NMR spectrum after the reaction of LSi(NHI) with $DippN_3$ in C_6D_6 at 298 K.

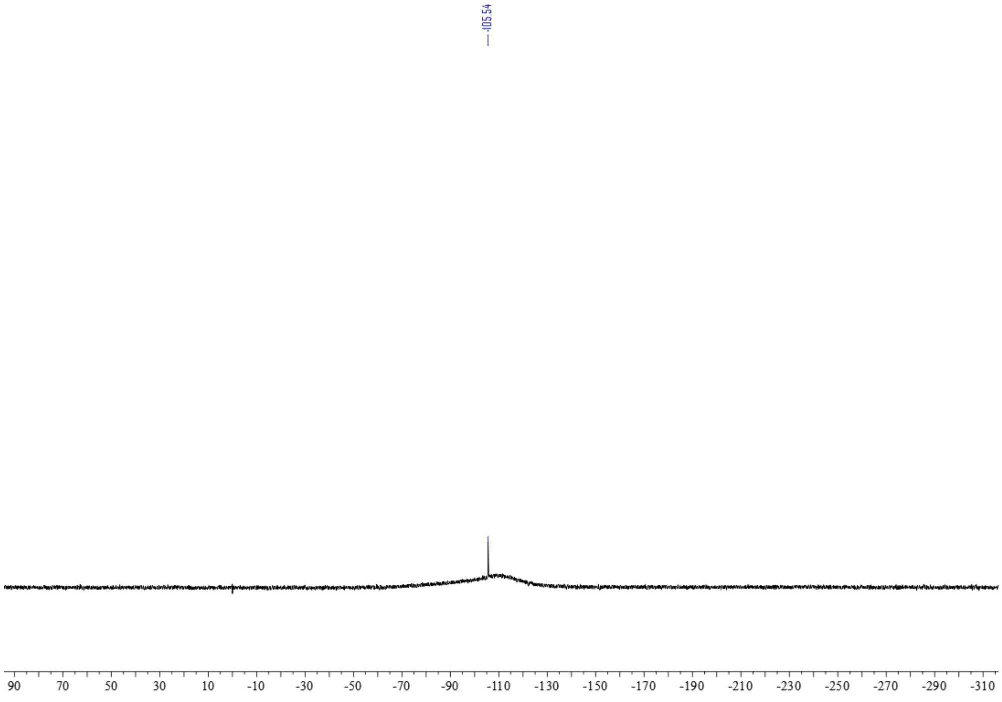

Supplementary Fig. 32. ^{29}Si NMR spectrum after the reaction of LSi(NHI) with $DippN_3$ in C_6D_6 at 298 K.

Reaction of 2 with 4,6-di-*tert*-butyl-N-(2,6-diisopropylphenyl)-*o*-iminobenzoquinone (imQ):

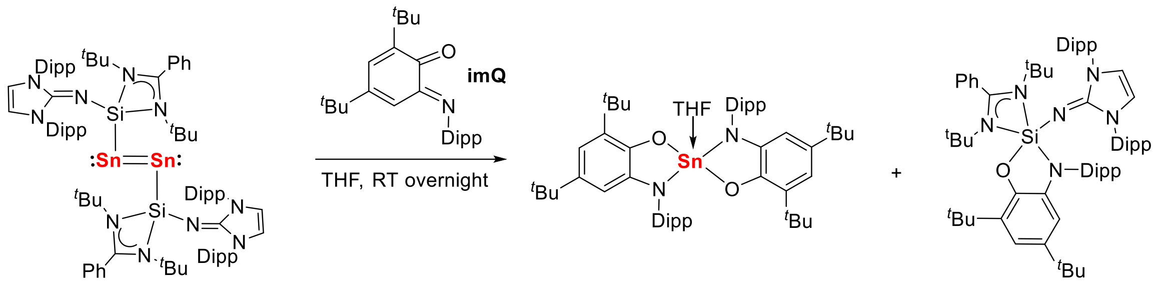

A THF solution (5 mL) of **2** (312 mg, 0.2 mmol) was added to the **imQ** (455.5 mg, 1.2 mmol) in THF (10 mL) over 10 mins at room temperature and the color was changed to the light brown. The solvent was removed in vacuum and subsequent NMR analysis revealed the clean formation of the five-coordinate bis(amidophenolato)tin(IV) complex along with a by-product (from the reaction of LSi(NHI) (L= PhC(N^{t}Bu)$_2$) with the **imQ:** See blow) and a little unknown compound with the ratio 1: 0.8 : 0.2. Characteristic NMR data of the five-coordinate bis(amidophenolato)tin(IV) complex: ^{1}H NMR spectrum of the compound are in agreement with reported literature[5].

^{1}H NMR (400 MHz, toluene-d_8, 298 K): δ 6.85 (d, 2H, 2.3 Hz, Ar*H*), 6.29 (d, 2H, 2.1 Hz, Ar*H*, *J*- (H-^{117,119}Sn) = 11.0 Hz), 4.05 (m, 2H$^{\alpha}$, CH_2 group of THF), 3.76 (m, 2H$^{\alpha}$, CH_2 group of THF), 3.40 (septet, 2H, 6.6 Hz, C*H*(CH_3)$_2$), 3.27 (septet, 2H, 6.4 Hz, C*H*(CH_3)$_2$), 1.34 (s, 18H, C(C*H*$_3$)$_3$), 1.19 (s, 18H, C(C*H*$_3$)$_3$), 1.03 (d, 6H, 6.9 Hz, CH(C*H*$_3$)$_2$). Several peaks for ArC, three sets of peaks for CH(C*H*$_3$)$_2$ and one set of peaks for H$^{\beta}$ of THF are overlapped with the peaks from the reaction of LSi(NHI) (L= PhC(N^{t}Bu)$_2$) with the **imQ**.

^{119}Sn{^{1}H} NMR (149 MHz, toluene-d_8, 298 K): δ -294.8.

^{29}Si{^{1}H} NMR (79 MHz, toluene-d_8, 298 K): δ -115.81.

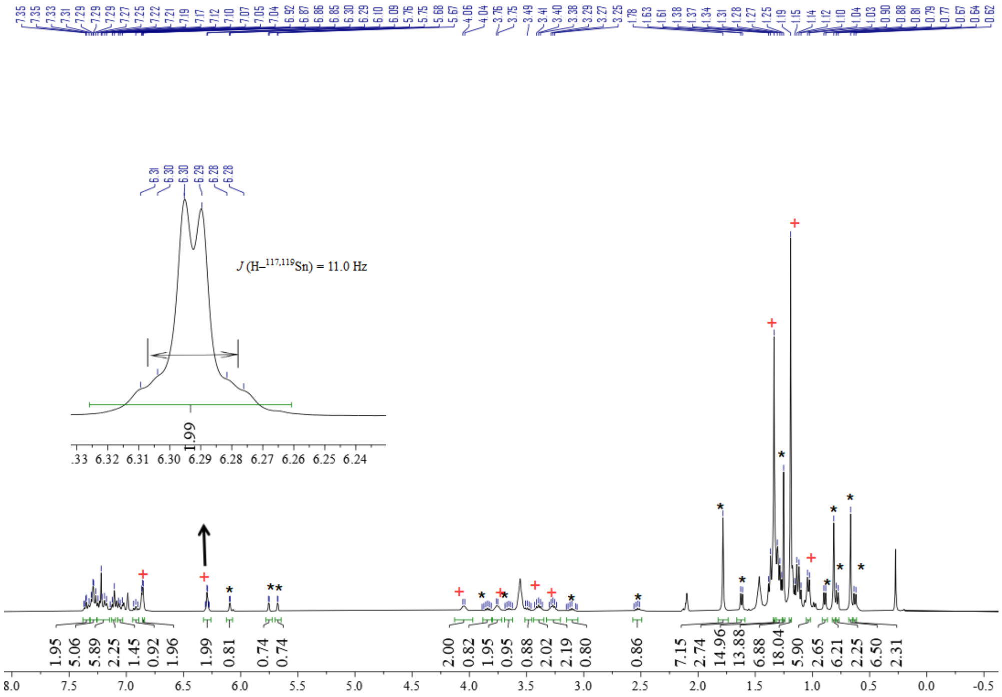

Supplementary Fig. 33. ^{1}H NMR spectrum after the reaction of **2** with **imQ** in toluene-d_8 at 298 K. Peaks with **+** are from the five-coordinate bis(amidophenolato)tin(IV) complex. Peaks with ***** are from the reaction of LSi(NHI) (L= PhC(N^{t}Bu)$_2$) with **imQ**.

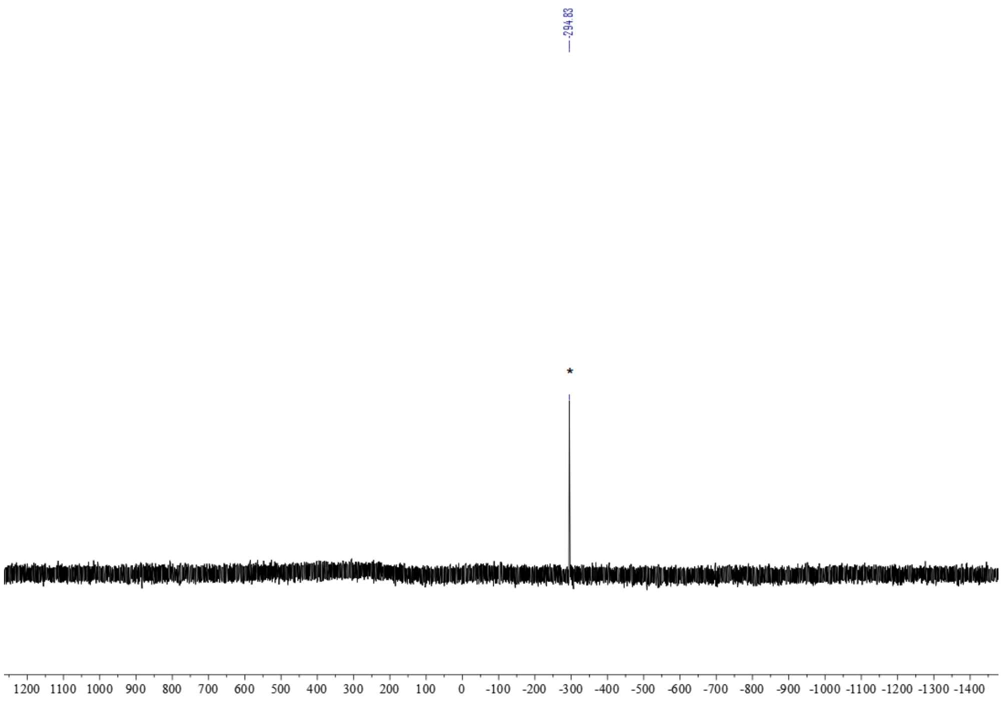

Supplementary Fig. 34. ^{119}Sn NMR spectrum after the reaction of **2** with **imQ** in toluene-d_8 at 298 K. Peak with ***** is from bis(amidophenolato)tin(IV) complex.

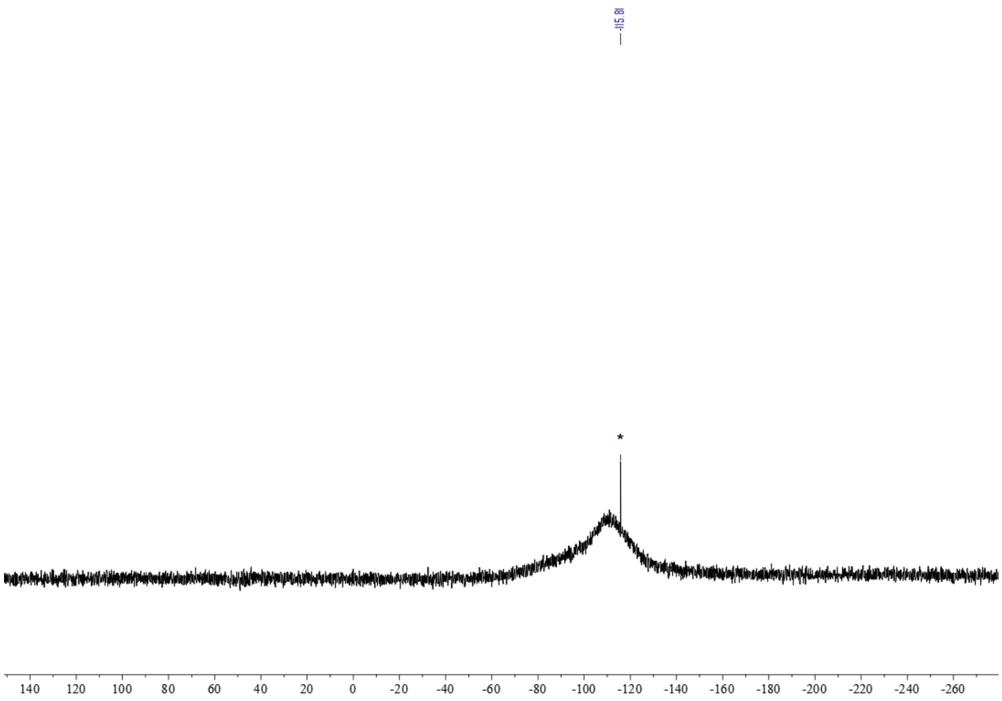

Supplementary Fig. 35. ^{29}Si NMR spectrum after the reaction of **2** with **imQ** in toluene-d_8 at 298 K. Peak with ***** is from the product of LSi(NHI) (L= PhC(NtBu)$_2$) with **imQ.**

Reaction of LSi(NHI) (L= PhC(N^{t}Bu)$_2$) with imQ:

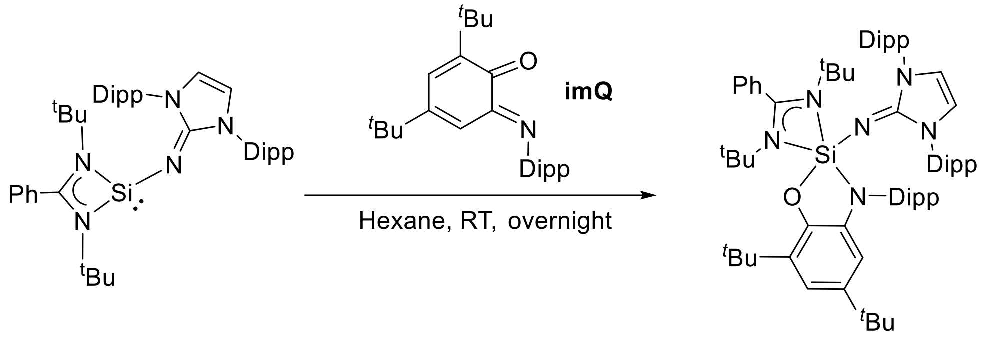

A hexane solution (2.0 mL) of LSi(NHI) (L= PhC(N^{t}Bu)$_2$) (662.1 mg, 1.0 mmol) was added to the **imQ** (379.6 mg, 1.0 mmol) in hexane (5.0 mL) dropwise at room temperature over a period of 2 minutes. After removal of the solvent, the resulting white solid was obtained in 97% yield.

^{1}H NMR (400 MHz, toluene-d$_8$, 298 K): δ 7.35-7.32 (m, 2H, Ar*H*), 7.29-7.26 (m, 2H, Ar*H*), 7.24-7.21 (m, 4H, Ar*H*), 7.07-7.05 (m, 2H, Ar*H*), 6.92-6.89 (m, 1H, Ar*H*), 6.85-6.84 (m, 2H, Ar*H*), 6.08 (d, *J* = 2.4 Hz, 1H, Ar*H*), 5.74 (d, *J* = 2.8 Hz, 1H, NC*H*), 5.66 (d, *J* = 1.6 Hz, 1H, NC*H*), 3.88-3.78 (m, 1H, C*H*(CH$_3$)$_2$), 3.69-3.61 (m, 1H, C*H*(CH$_3$)$_2$), 3.58-3.43 (m, 2H, C*H*(CH$_3$)$_2$), 3.14-3.04 (m, 1H, C*H*(CH$_3$)$_2$), 2.56-2.47 (m, 1H, C*H*(CH$_3$)$_2$), 1.77 (s, 9H, C(C*H*$_3$)$_3$), 1.61 (d, *J* = 6.4 Hz, 3H, CH(C*H*$_3$)$_2$), 1.36 (d, *J* = 6.8 Hz, 3H, CH(C*H*$_3$)$_2$), 1.14-1.09 (m, 12H, CH(C*H*$_3$)$_2$), 0.88 (d, *J* = 5.2 Hz, 3H, CH(C*H*$_3$)$_2$), 0.65 (s, 9H, C(C*H*$_3$)$_3$), 0.61 (d, *J* = 6.4 Hz, 3H, CH(C*H*$_3$)$_2$). Several peaks for Ar*C* are overlapped with the solvent residual signal of toluene-d$_8$ and Several peaks for CH(C*H*$_3$)$_2$ are partially overlapped with the one peak for C(C*H*$_3$)$_3$.

^{13}C{^{1}H} NMR (400 MHz, toluene-d$_8$, 298 K): δ 170.2 (s, N*C*N), 150.2 (s, Ar*C*), 149.4 (s, Ar*C*), 149.2 (s, Ar*C*), 148.3 (s, Ar*C*), 147.7 (s, Ar*C*), 145.0 (s, Ar*C*), 143.4 (s, Ar*C*), 143.2 (s, Ar*C*), 140.2 (s, Ar*C*), 138.3 (s, Ar*C*), 137.6 (s, Ar*C*), 137.1 (s, Ar*C*), 135.9 (s, Ar*C*), 131.5 (s, Ar*C*), 130.2 (s, Ar*C*), 129.4 (s, Ar*C*), 129.3 (s, Ar*C*), 128.8 (s, Ar*C*), 128.4 (s, Ar*C*), 127.5 (s, Ar*C*), 127.0 (s, Ar*C*), 126.8 (s, Ar*C*), 125.7 (s, Ar*C*), 124.8 (s, Ar*C*), 124.5 (s, Ar*C*), 124.4 (s, Ar*C*),

124.2 (s, Ar*C*), 123.0 (s, Ar*C*), 116.9 (s, Ar*C*), 116.3 (s, Ar*C*), 112.5 (s, Ar*C*), 109.7 (s, Ar*C*), 53.80 (s, *C*$(CH_3)_3$), 52.75 (s, *C*$(CH_3)_3$), 34.94 (s, *C*$(CH_3)_3$), 34.52 (s, *C*$(CH_3)_3$), 33.51 (s, *C*H$(CH_3)_2$), 32.82 (s, *C*H$(CH_3)_2$), 32.00 (s, *C*H$(CH_3)_2$), 31.78 (s, *C*H$(CH_3)_2$),, 29.13 (s, *C*H$(CH_3)_2$), 28.88 (s, *C*H$(CH_3)_2$), 28.63 (s, CH_3), 28.62 (s, CH_3), 28.18 (s, CH_3), 28.11 (s, CH_3), 27.55 (s, CH_3), 27.49 (s, CH_3), 26.90 (s, CH_3), 26.69 (s, CH_3), 26.44 (s, CH_3), 26.28 (s, CH_3), 24.92 (s, CH_3), 24.74 (s, CH_3), 24.37 (s, CH_3), 23.22 (s, CH_3), 23.09 (s, CH_3).one peak for CH_3 is overlapped with the solvent residual signal of toluene-d_8.

$^{29}Si\{^1H\}$ NMR (79 MHz, toluene-d_8, 298 K): δ -115.81.

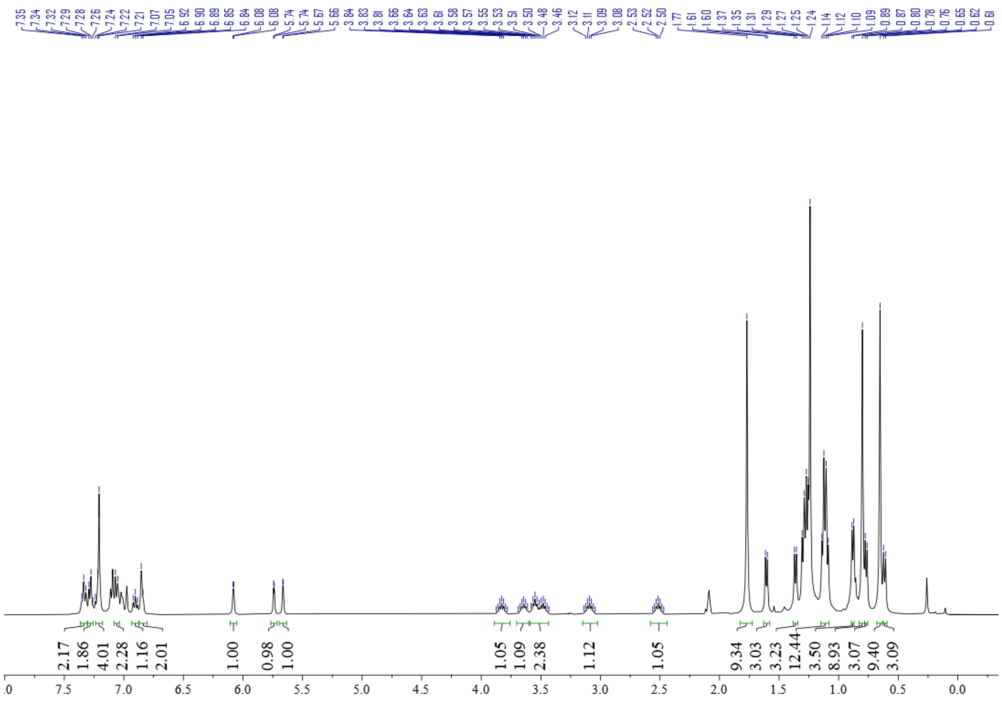

Supplementary Fig. 36. ^{1}H NMR spectrum after the reaction of LSi(NHI) with **imQ** in toluene-d_8 at 298 K.

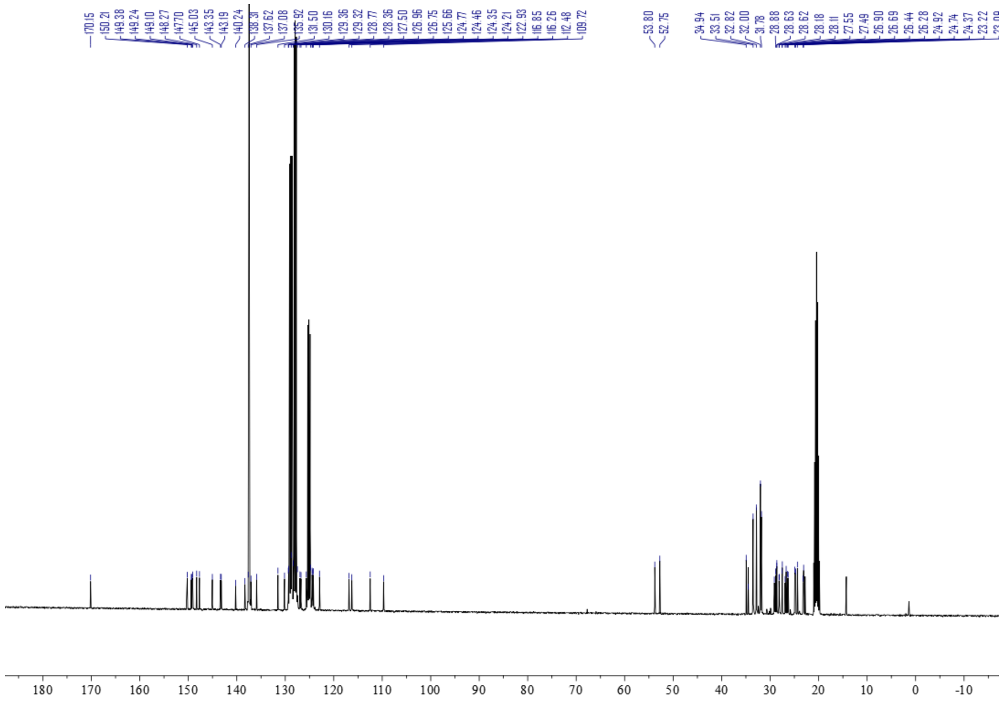

Supplementary Fig. 37. ^{13}C NMR spectrum after the reaction of LSi(NHI) with **imQ** in toluene-d_8 at 298 K.

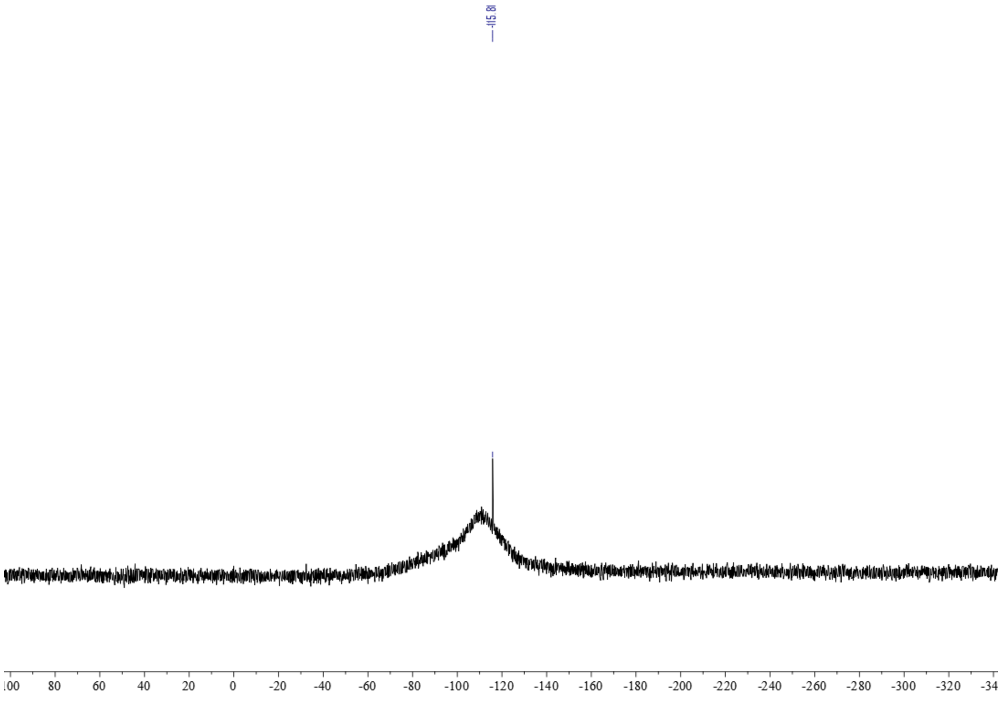

Supplementary Fig. 38. ^{29}Si NMR spectrum after the reaction of LSi(NHI) with **imQ** in toluene-d_8 at 298 K.

Preparation of IPr→$SnBr_2$:

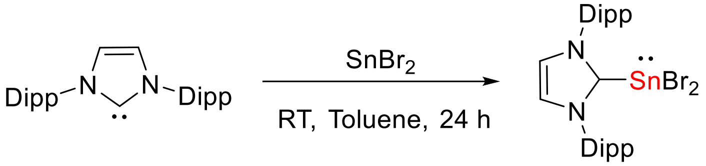

A toluene suspension (10 mL) of $SnBr_2$ (418 mg, 1.5 mmol) was added to the IPr (661 mg, 1.7 mmol) in toluene (10 mL) over 15 min at room temperature. After stirring for 48 h at this temperature, a white slurry was obtained. Then the solution was concentrated to 8 mL and the white precipitate was isolated by filtration to yield the **IPr-$SnBr_2$** in 85% yield. Single crystals suitable for X-ray diffraction studies were obtained by vapor diffusion of *n*-pentane into the THF solution at room temperature.

^{1}H NMR (400 MHz, C_6D_6, 298 K): δ 7.23 (t, *J* = 7.6 Hz, 2H, Ar*H*), 7.08 (d, *J* = 8.0 Hz, 4H, Ar*H*), 6.47 (s, 2H, NC*H*), 2.85-2.75 (m, 4H, C*H*(CH_3)$_2$), 1.42 (d, *J* = 6.8 Hz, 12H, CH(CH_3)$_2$), 0.98 (d, *J* = 6.8 Hz, 12H, CH(CH_3)$_2$).

^{13}C{^{1}H} NMR (101 MHz, C_6D_6, 298 K): δ 145.9 (s, Ar*C*), 133.7 (s, Ar*C*), 131.4 (s, N-*C*H-), 124.8 (s, Ar*C*), 124.6 (s, Ar*C*), 29.21 (s, *C*H(CH_3)$_2$), 25.84 (s, *C*H_3), 23.38(s, *C*H_3).

^{119}Sn{^{1}H} NMR (149 MHz, C_6D_6, 298 K): δ -21.3 (s).

Anal. Calcd for $C_{27}H_{36}Br_2N_2Sn$: C, 48.61; H, 5.44; N, 4.20. Found: C, 48.43; H, 5.36; N, 4.14.

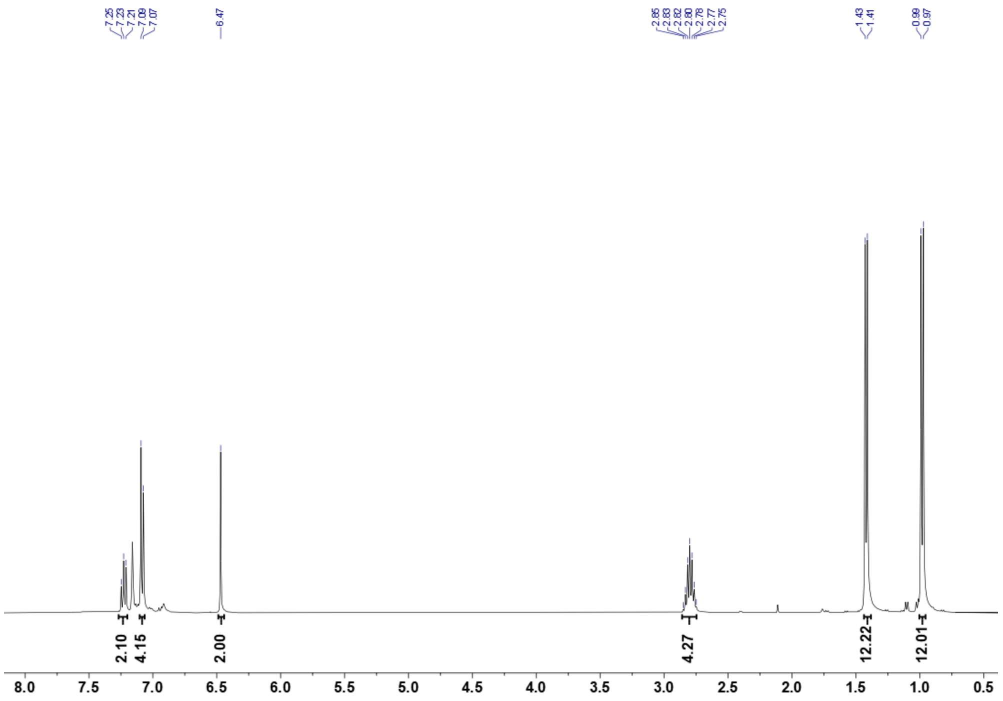

Supplementary Fig. 39. ^{1}H NMR spectrum of **IPr-SnBr$_2$** in C_6D_6 at 298 K.

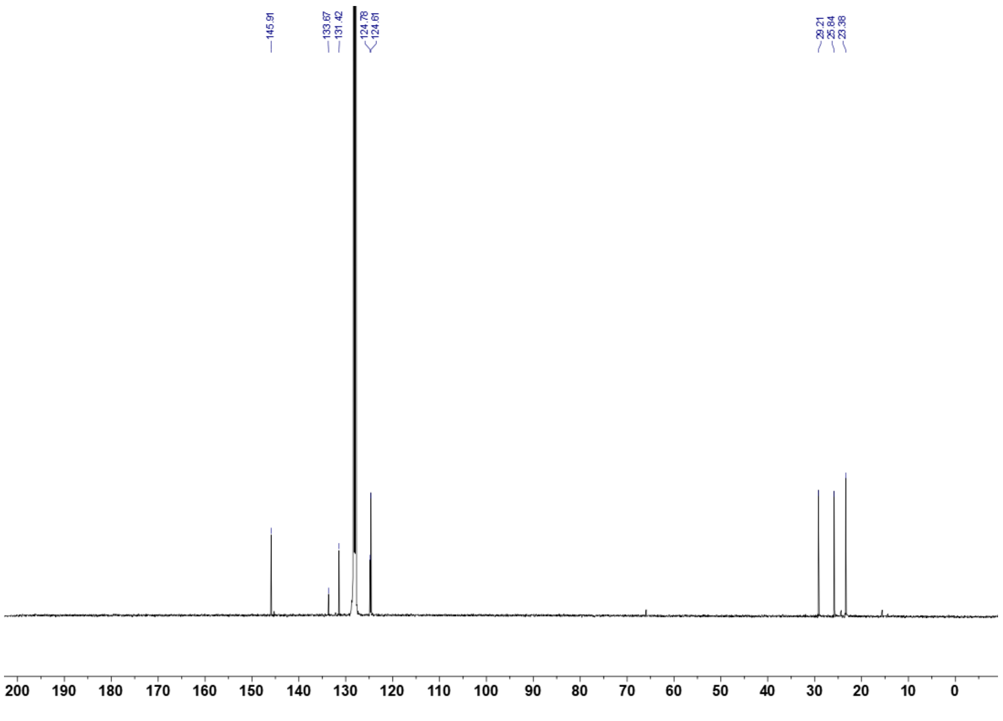

Supplementary Fig. 40. ^{13}C NMR spectrum of **IPr-SnBr$_2$** in C_6D_6 at 298 K.

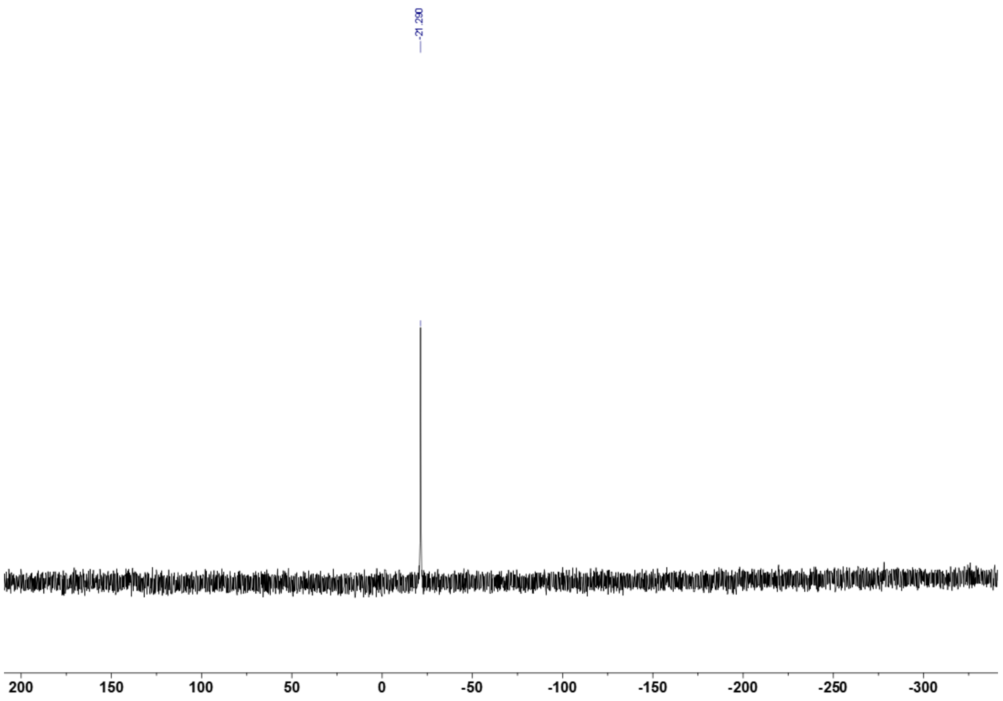

Supplementary Fig. 41. ^{119}Sn NMR spectrum of **IPr-SnBr$_2$** in C_6D_6 at 298 K.

2.Supplementary Discussion

X-ray Crystallographic Studies

The CCDC numbers 2254817, 2254818, 2254819, and 2254820, 2254821 contain the supplementary crystallographic data for this paper. These data can be obtained free of charge by contacting The Cambridge Crystallographic Data Centre.

Crystals for X-ray diffraction studies were obtained as described in the preparations. The crystals were manipulated in a glovebox under a microscope, and were sealed in thin-walled glass capillaries. Crystals for X-ray diffraction studies were obtained as described in the preparations. The X-ray crystallographic data for compounds **1, 2, 3, 4,** and **5** were collected with a Rigaku Saturn 724 CCD diffractometer using graphite-monochromated Mo Kα radiation (λ = 0.71073 Å) at 113(2) K. The structures were solved with the Olex2 and refined with the ShelXL refinement package using Least Squares minimization[6-8]. Refinement was performed on F^2 anisotropically for all the non-hydrogen atoms by the full-matrix least-squares method. The hydrogen atoms were placed at the calculated positions and were included in the structure calculation without further refinement of the parameters. The Olex2 program was utilized to draw the molecular structures[6]. Details of the crystallographic data and a summary of the intensity data collection parameters for **1, 2, 3, 4,** and **5** are listed in Supplementary Tab. 1 and Supplementary Tab. 2. Molecular structure of **1, 2, 3, 4,** and **5** are shown in Supplementary Supplementary Fig. 42-46.

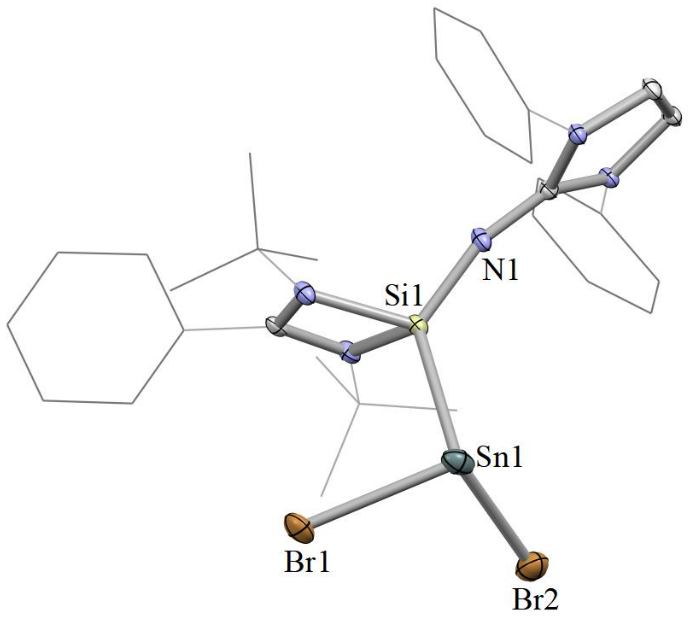

Supplementary Fig. 42. X-ray crystal structure of **1** at 30% probability ellipsoids. Hydrogen atoms and ^{i}Pr groups are omitted for clarity. Selected bond lengths (Å) and angles (º): Si1−Sn1 2.725(1), Si1−N1 1.637(3), Sn1−Br1 2.6225(6), Sn1−Br2 2.6493(6), Sn1−Si1−N1 121.4(1).

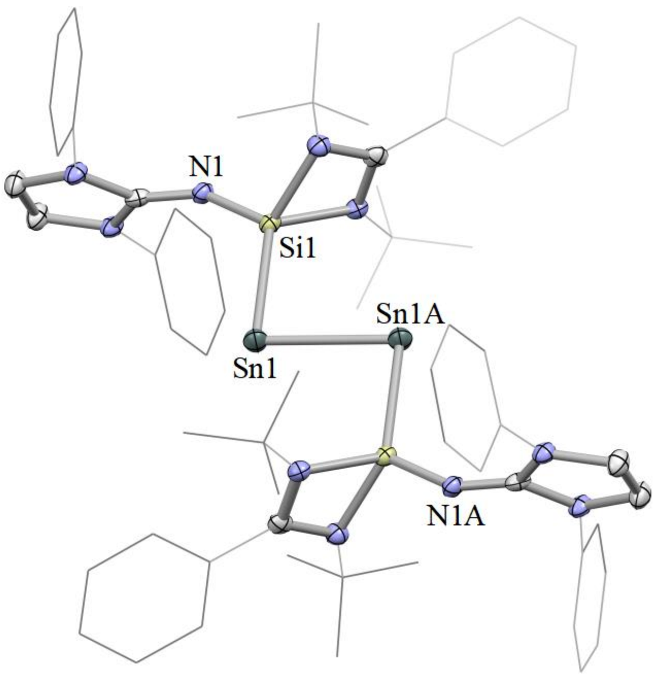

Supplementary Fig. 43. X-ray crystal structure of **2** at 30% probability ellipsoids. Hydrogen atoms and ^{i}Pr groups are omitted for clarity. Selected bond lengths (Å) and angles (°): Sn1−Sn1A 2.7240(6), Si1−Sn1 2.607(1), Si1−N1 1.673(3), Sn1−Si1−N1 121.1(1), Si1−Sn1−Sn1A 89.20(3).

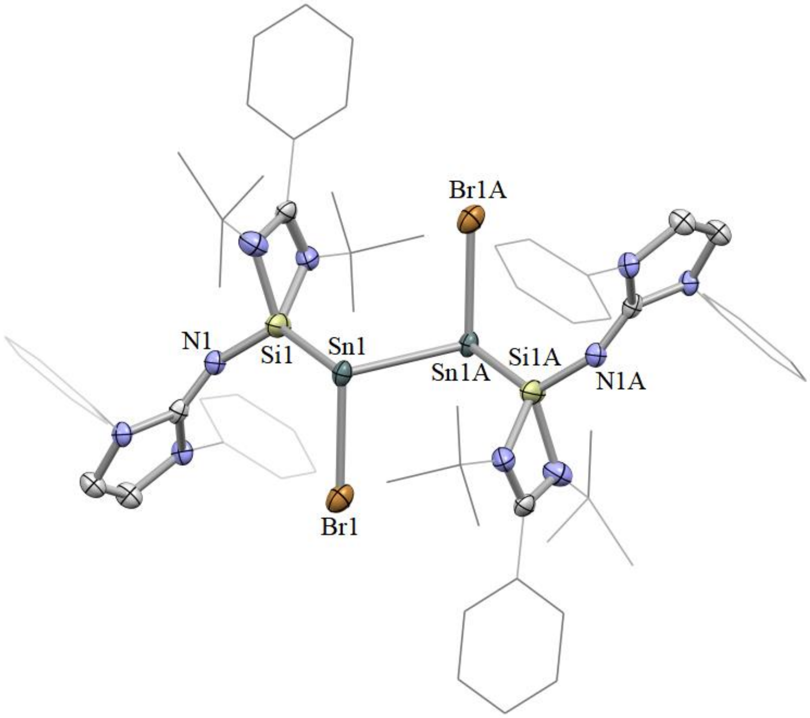

Supplementary Fig. 44. X-ray crystal structure of **3** at 30% probability ellipsoids. Hydrogen atoms and ^{i}Pr groups are omitted for clarity. Selected bond lengths (Å) and angles (°): Sn1−Sn1A 2.9138(6), Si1−Sn1 2.756(2), Si1−N1 1.621(3), Sn1−Br1 2.6749(7), Sn1−Si1−N1 121.9(1), Si1−Sn1−Sn1A 89.76(3), Si1−Sn1−Br1 93.75(4).

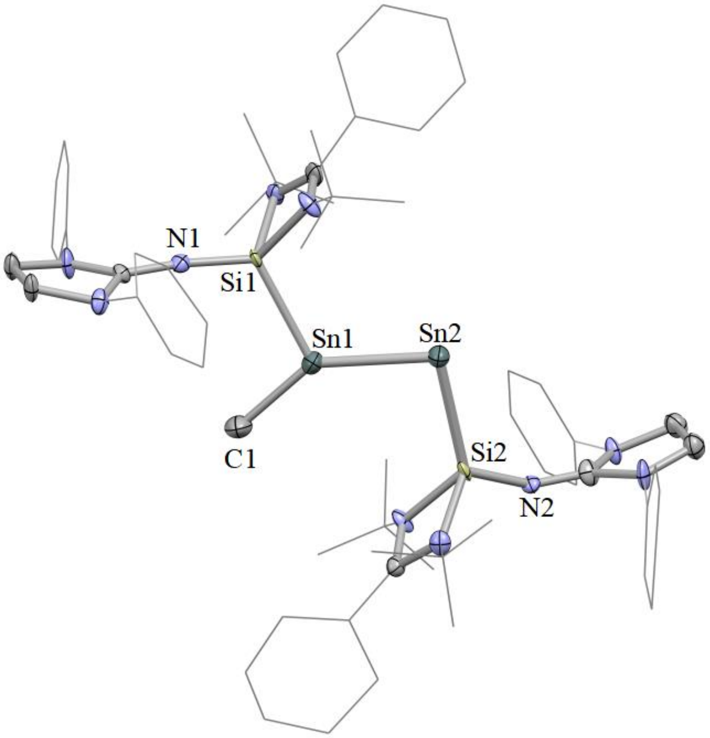

Supplementary Fig. 45. X-ray crystal structure of **4** at 30% probability ellipsoids. Hydrogen atoms and ^{i}Pr groups are omitted for clarity. Selected bond lengths (Å) and angles (°): Sn1−Sn2 2.646(1), Si1−Sn1 2.512(7), Si2−Sn2 2.630(8), Sn1−C1 2.186(8), Si1−Sn1−Sn2 113.2(2), Sn1−Sn2−Si2 96.2(2), Si1−Sn1−C1 105.5(3), C1−Sn1−Sn2 140.8(2), Si1−Sn1−Sn2−Si2 172.0(3).

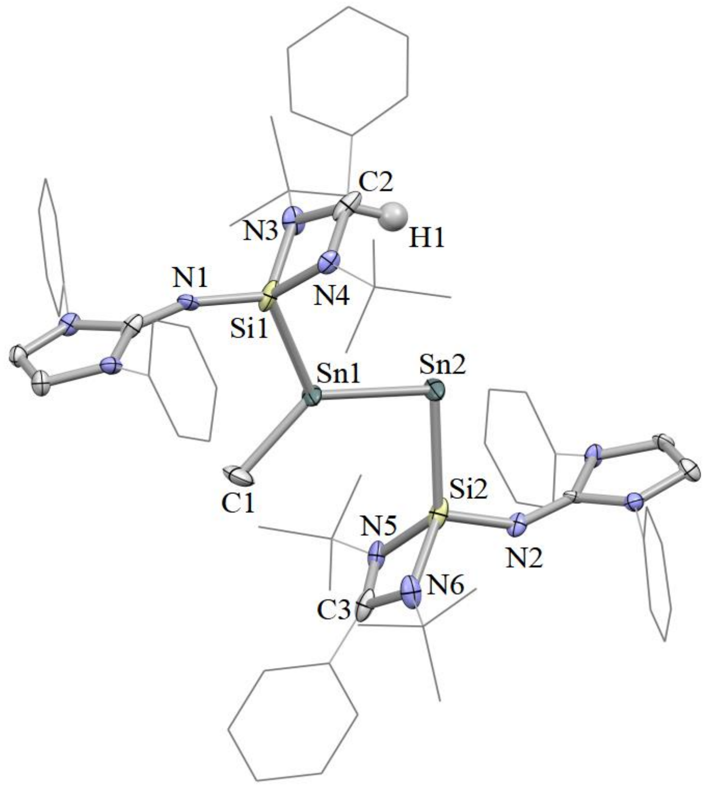

Supplementary Fig. 46. X-ray crystal structure of **5** at 30% probability ellipsoids. Hydrogen atoms and ^{i}Pr groups are omitted for clarity. Selected bond lengths (Å) and angles (°): Sn1−Sn2 2.654(1), Si1−Sn1 2.537(4), Si2−Sn2 2.676(4), Sn1−C1 2.16(2), Si1−N1 1.67(1), Si2−N2 1.64(1), C2−H1 1.00, C2−N3 1.46(2), C2−N4 1.48(2), C3−N5 1.35(2), C3−N6 1.40(1), Si1−Sn1−Sn2 119.04(9). Sn1−Sn2−Si2 97.79(9), Si1−Sn1−C1 110.9(4), C1−Sn1−Sn2 130.0(4), Si1−Sn1−Sn2−Si2 175.7(1).

Supplementary Tab. 1. X-Ray crystallographic data and structure refinement for 1, 2 and 3.

	**1**	**2**	**3**
formula	$C_{42}H_{59}Br_2N_5SiSn$	$C_{84}H_{118}N_{10}Si_2Sn_2$	$C_{96}H_{130}Br_2N_{10}Si_2Sn_2$
formula weight	940.54	1561.44	1877.47
crystal system	triclinic	monoclinic	monoclinic
space group	P-1	I2/a	I2/a
a, Å	10.5751(4)	23.2774(6)	24.7568(7)
b, Å	12.2646(5)	18.8688(6)	18.8238(5)
c, Å	17.4458(6)	24.8648(8)	24.6483(6)
α, deg	90.060(3)	90	90
β, deg	90.862(3)	101.263(3)	103.523(3)
γ, deg	104.104(3)	90	90
V, Å^3	2194.23(15)	10710.7(6)	11168.1(5)
Z	2	4	4
D_{calcd}, g/cm^3	1.424	0.968	1.117
temp, K	113	113	113
m, mm^{-1}(MoKa)	0.71073	0.71073	0.71073
reflections collected	23067	38624	40461
independent reflections (R_{int})	8975（0.0535）	10922 (0.0461)	11341 (0.0800)
$R1$ ($I > 2\sigma(I)$)	0.0476	0.0554	0.0571
$wR2$ ($I > 2\sigma(I)$)	0.1143	0.1516	0.1387
$wR2$ (all data)	0.1236	0.1605	0.1564
parameters	474	456	621
GOF	1.031	1.036	1.043

Supplementary Tab. 2. X-Ray crystallographic data and structure refinement for 4, 5 and IPr-SnBr2.

	**4**	**5**
formula	$C_{117}H_{133}BF_{24}N_{10}Si_2Sn_2$	$C_{93}H_{142}N_{10}O_2Si_2Sn_2$
formula weight	2439.70	1725.72
crystal system	monoclinic	monoclinic
space group	C2/c	Pn
a, Å	18.7579(4)	14.4510(2)
b, Å	18.8399(4)	21.8564(3)
c, Å	37.9248(7)	15.5924(2)
α, deg	90	90
β, deg	101.584(2)	101.797(2)
γ, deg	90	90
V, Å^3	13129.5(5)	4820.79(12)
Z	4	2
D_{calcd}, g/cm^3	1.234	1.189
temp, K	113	113
m, mm^{-1}(MoKa)	0.71073	0.71073
reflections collected	57315	39246
independent reflections (R_{int})	13438 (0.0526)	19502 (0.0717)
$R1$ ($I > 2\sigma(I)$)	0.0642	0.0832
$wR2$ ($I > 2\sigma(I)$)	0.1722	0.1855
$wR2$ (all data)	0.1803	0.1906
parameters	1219	1023
GOF	1.073	1.078

Computational studies

All DFT calculations were carried out with Gaussian16-C.01 quantum chemical package.[9] Geometry optimizations were performed in the gas phase with the PBE0/Def2TZVP level of theory on **2, 4** and **5**. Vibrational frequency calculations were carried out at the same level of theory as the geometry optimizations (No imaginary frequency for local minima) and to provide the thermal corrections for Gibbs free energy determinations at 298.15 K and 1atm. The UV-vis spectrum of **2** and **5** were calculated using TD-PBE0 method at def2SVP basis set. Intrinsic reaction coordinate (IRC) calculations were done to confirm that the transition states proposed connected the appropriate reactants and products. The single-point calculations of the optimized geometries were performed with PBE0 functional with Grimme's D3BJ dispersion correction and triple-zeta quality Def2TZVP basis set[10-11]. Natural Bond Orbital (NBO) analyses were performed using the NBO 7.0 program at PBE0/Def2SVP level[12]. Wiberg bond indexes were determined from the Natural Atomic Orbital basis. Electronic wavefunction analysis is performed by Multiwfn package[13-14]. Graphical structures are visualized with VMD[15] and CYLview.[16]

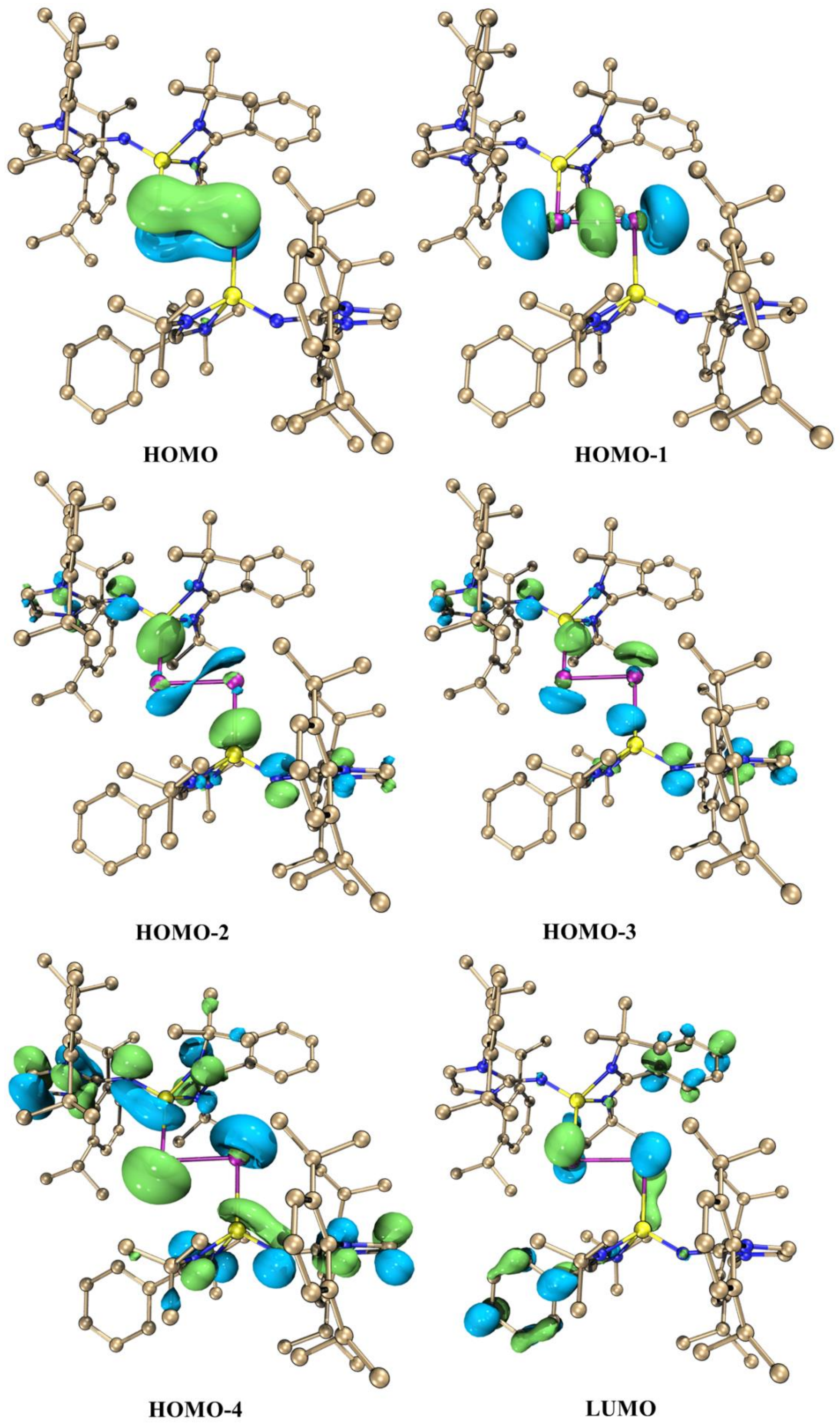

Supplementary Fig. 47. Selected Kohn-Sham isosurfaces (0.05 au) in **2**.

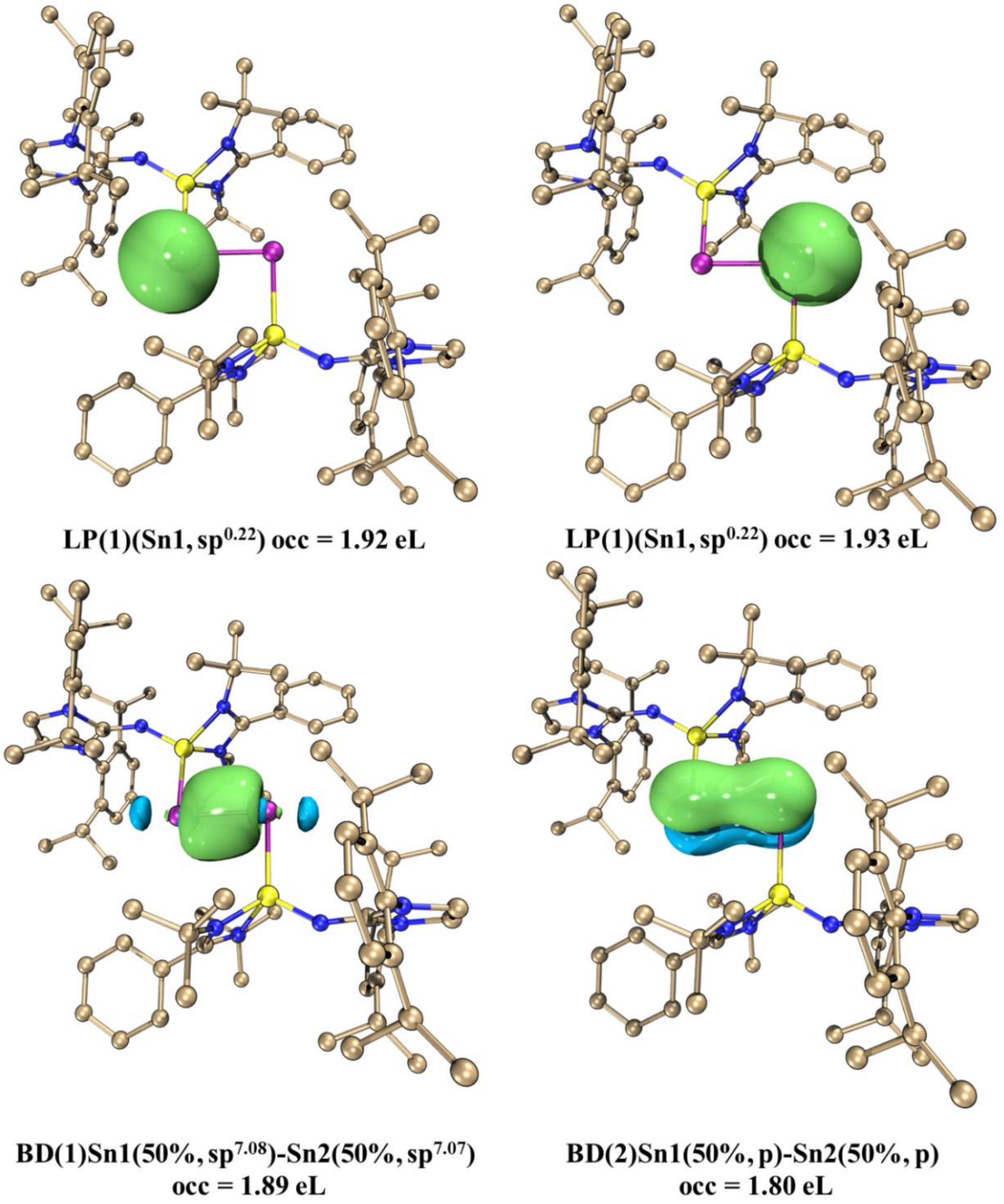

Supplementary Fig. 48. Selected NBOs (0.05 au) in **2**. (LP = lone pair of electrons, BD = 2-center bond, occ = occupancy).

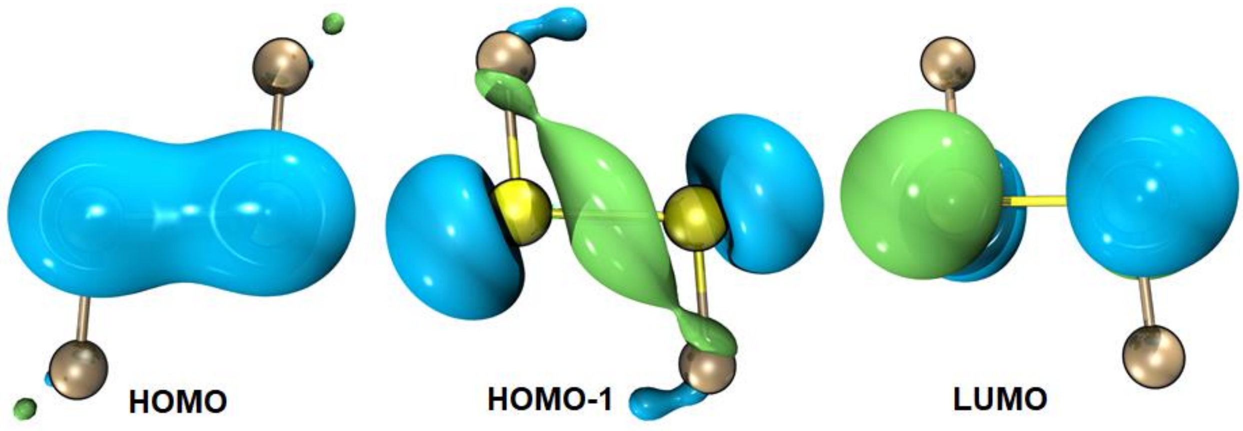

Supplementary Fig. 49. Selected Kohn-Sham isosurfaces (0.05 au) in MeSb=SbMe.

Supplementary Tab. 3. Comparison of computed and experimental structure of compound **2**

Selected bond lengths (Å) and angles (º)	Computed structure	Experimental structure	Deviation(%)
Sn1-Sn1A	2.717	2.7240(6)	0.25
Si1-Sn1	2.606	2.607(1)	0.038
Si1-N1	1.689	1.673(3)	0.95
Si1A-Sn1A	2.611	2.607(1)	0.15
Si1A-N1A	1.690	1.673(3)	1
Sn1-Si1-N1	119.238	121.1(1)	1.6
Sn1A-Si1A-N1A	118.862	121.1(1)	1.8
Si1-Sn1-Sn1A	87.519	89.20(3)	1.9
Sn1A-Sn1A-Sn1	88.048	89.20(3)	1.3

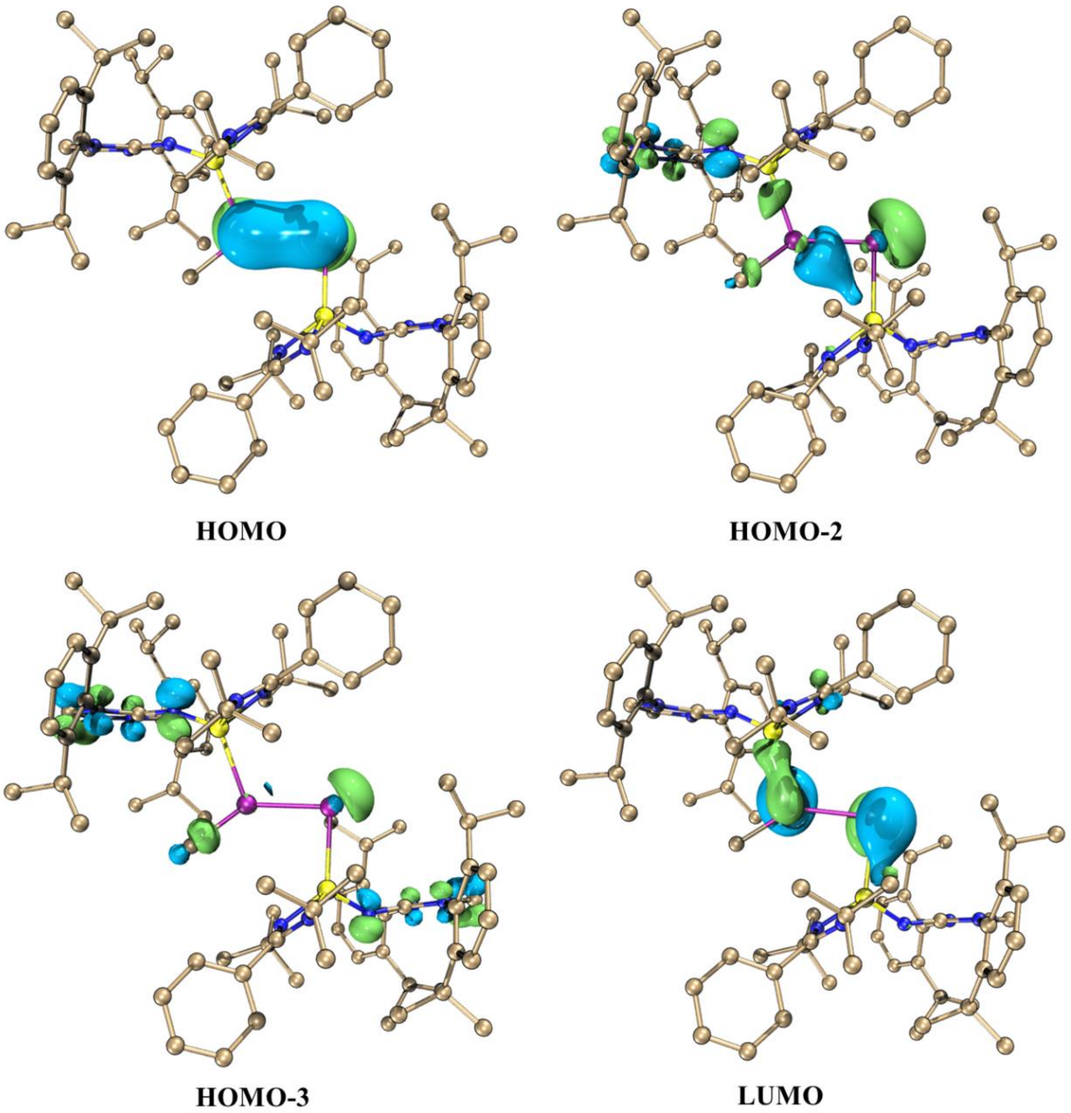

Supplementary Fig. 50. Selected Kohn-Sham isosurfaces (0.05 au) in **4**.

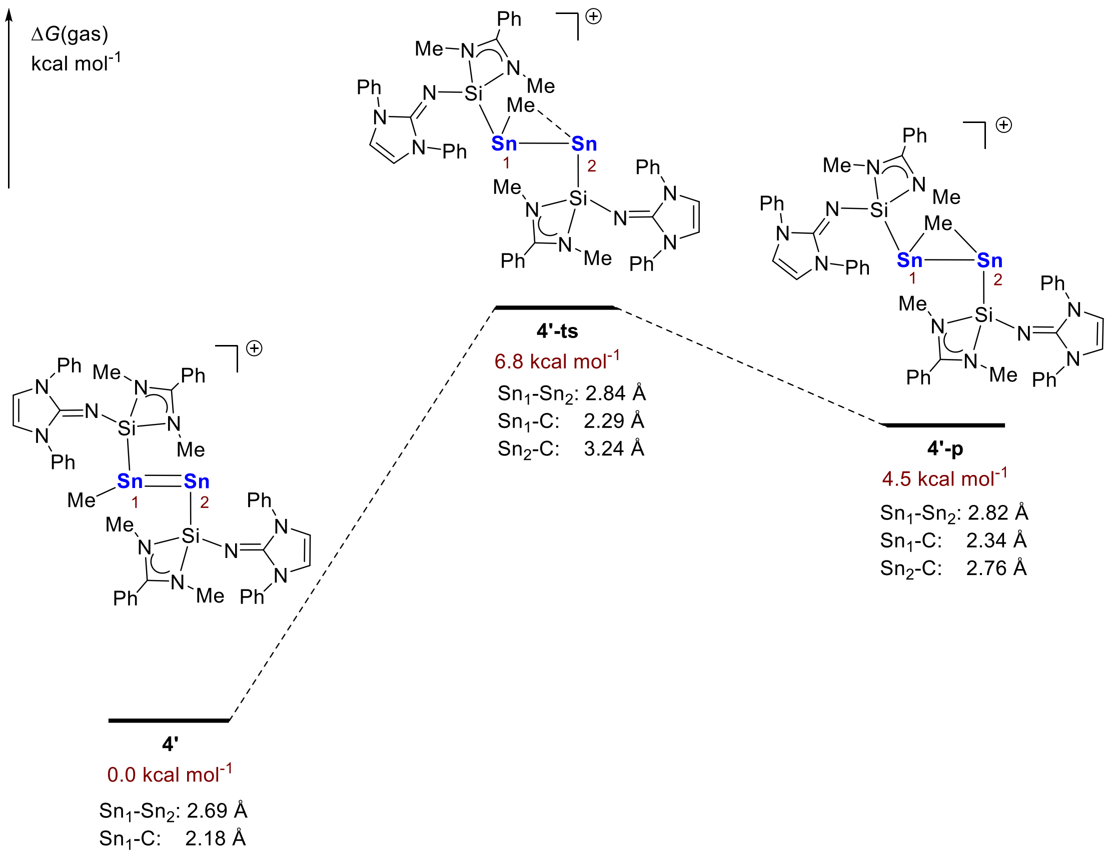

Supplementary Fig. 51. Schematic Gibbs profiles (T = 298 K) for the degenerate isomerization of a model system **4**

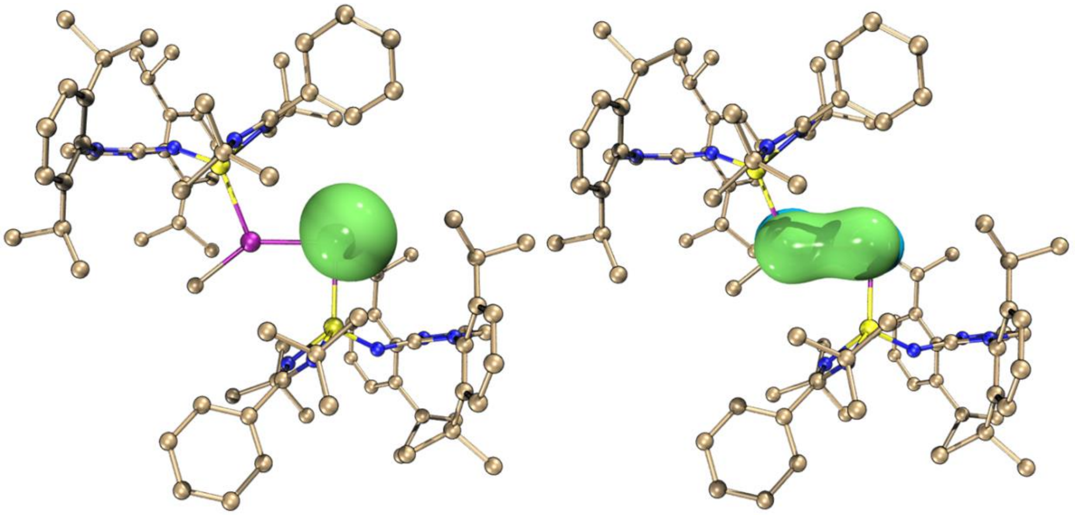

LP(1)(Sn1, $sp^{0.18}$) occ = 1.90 eL   BD(1)Sn1(46%, $sp^{0.73}$)-Sn2(54%, $sp^{14.12}$) occ = 1.86 eL

Supplementary Fig. 52. Selected NBOs (0.05 au) in **4**. (LP = lone pair of electrons, BD = 2-center bond, occ = occupancy).

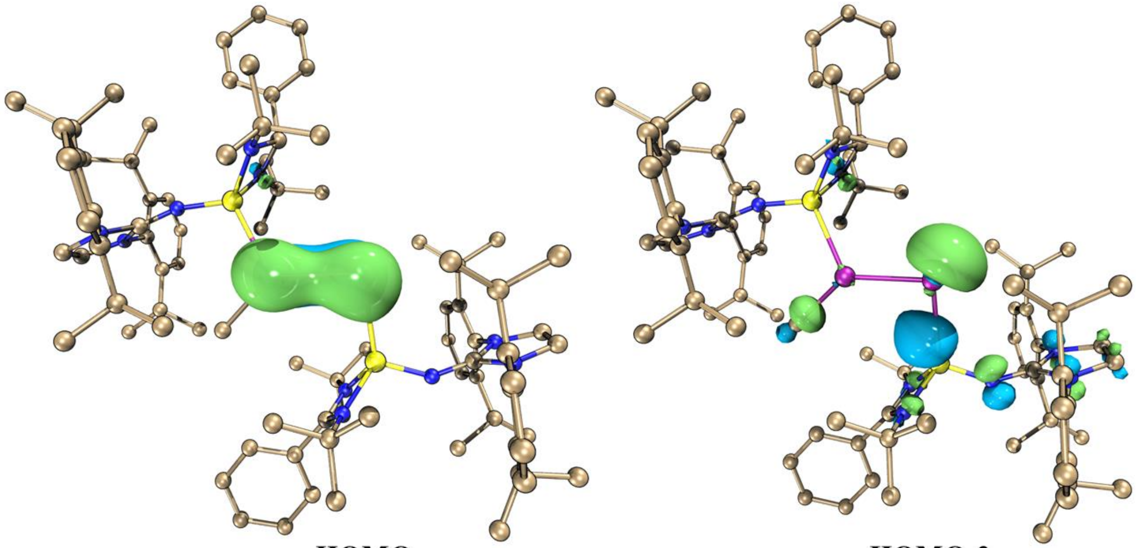

HOMO   HOMO-2

Supplementary Fig. 53. Selected Kohn-Sham isosurfaces (0.05 au) in **5**.

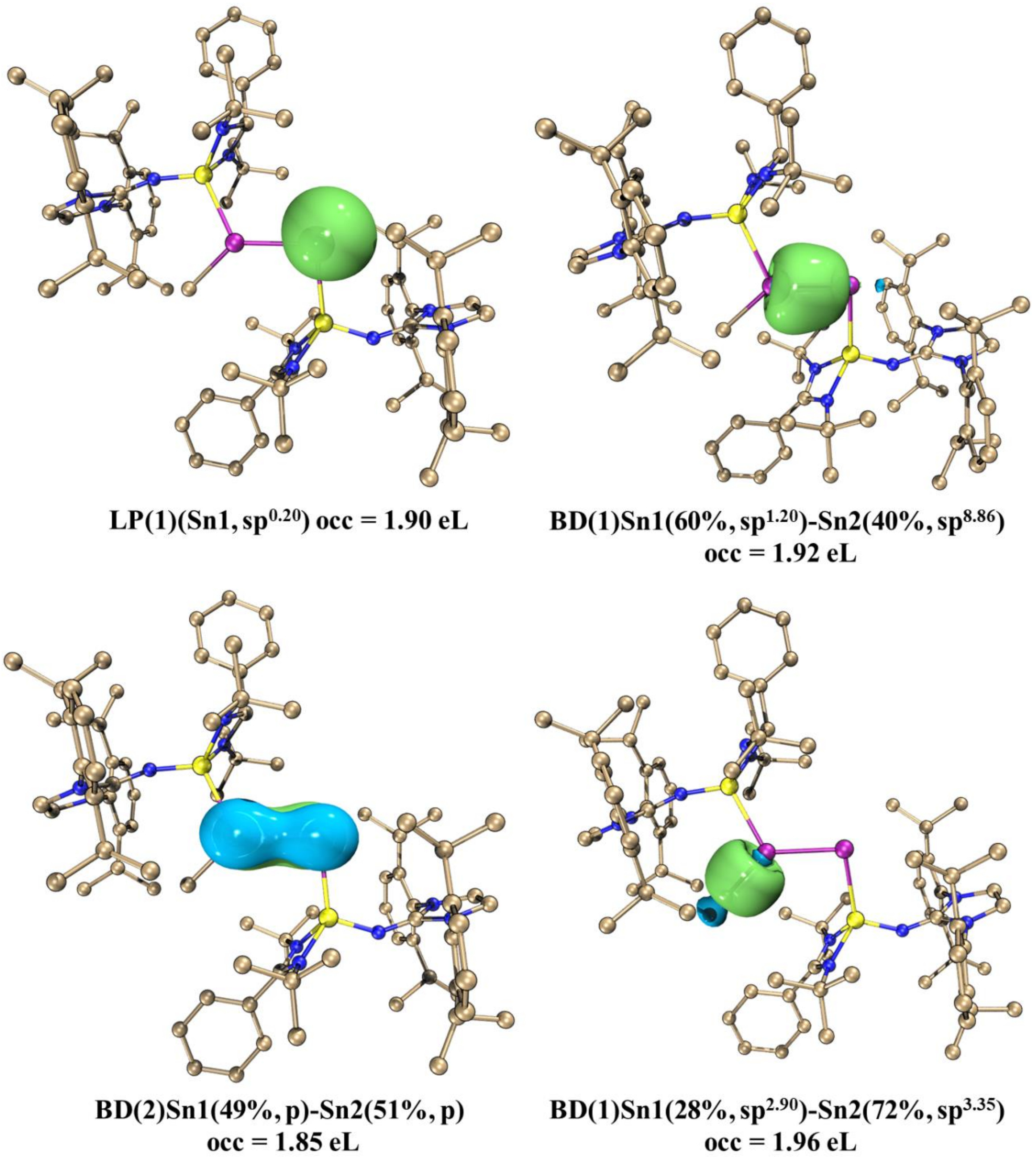

Supplementary Fig. 54. Selected NBOs (0.05 au) in **5**. (LP = lone pair of electrons, BD = 2-center bond, occ = occupancy).

Selected TDDFT outputs:

Ditin(0) 2

Excitation energies and oscillator strengths:

Excited State 1: Singlet-A 1.8520 eV 669.45 nm f=0.0001 <S**2>=0.000

381 -> 383 0.69835

This state for optimization and/or second-order correction.

Total Energy, E(TD-HF/TD-DFT) = -4819.66387962

Copying the excited state density for this state as the 1-particle RhoCI density.

Excited State 2: Singlet-A 2.0471 eV 605.66 nm f=0.1236 <S**2>=0.000

382 -> 383 0.69178

Distannavinylidene 5

Excitation energies and oscillator strengths:

Excited State 1: Singlet-A 2.2879 eV 541.91 nm f=0.2470 <S**2>=0.000

386 -> 388 0.13402

387 -> 388 0.68285

This state for optimization and/or second-order correction.

Total Energy, E(TD-HF/TD-DFT) = -4860.07674825

Copying the excited state density for this state as the 1-particle RhoCI density.

References

(1) Pompeo, M., Froese, R. D. J., Hadei, N. & Organ, M. G. Pd-PEPPSI-IPENT^{Cl}: a highly effective catalyst for the selective cross-coupling of secondary organozinc reagents. *Angew. Chem. Int. Ed.* **51**, 11354-11357 (2012).

(2) Du, S. et al. Synthesis and reactivity of N-heterocyclic silylene stabilized disilicon(0) complexes. *Angew. Chem. Int. Ed.* **61**, e202115570 (2022).

(3) Hicks, J., Juckel, M., Paparo, A., Dange, D. & Jones, C. Multigram syntheses of magnesium(I) compounds using alkali metal halide supported alkali metals as dispersible reducing agents. *Organometallics* **37**, 4810-4813 (2018).

(4) Merrill, W. A. et al. Synthesis and characterization of the unstable primary amido tin(II) dimer $Sn_2\{N(H)Dipp\}_4$ (Dipp = C_6H_3-2,6-$Pr^i{}_2$) and the first sesqui-amido hemi-chloride derivative $Sn_2\{N(H)Dipp\}_3Cl$: facile conversion of a primary amide to the imide $(SnNDipp)_4$. *Dalton. Trans.* 5905-5910 (2008).

(5) Piskunov, A. V. et al. New tin(II) and tin(IV) amidophenolate complexes. *Inorg. Chem. Commun.* **9**, 612-615 (2006).

(6) Dolomanov, O. V., Bourhis, L. J., Gildea, R. J., Howard, J. A. K. & Puschmann, H. Olex2: a complete structure solution, refinement and analysis program. *J. Appl. Crystallogr.* **42**, 339-341 (2009).

(7) Sheldrick, G. M., SHELXS-90/96, Program for Structure Solution, Acta Crystallogr. Sect. A, **46**, 467 (1990).

(8) Sheldrick, G. M., SHELXL 97, *Program for Crystal Structure Refinement*, University of Goettingen, Goettingen,Germany, (1997).

(9) Frisch, MJ. Trucks GW., Schlegel HB., Scuseria GE, Robb MA, Cheeseman JR., Scalmani G, Barone V, Petersson GA, Nakatsuji H, Caricato X. Li, M., Marenich AV., Bloino J, Janesko BG, Gomperts R., Mennucci B, Hratchian HP, Ortiz JV, Izmaylov AF, Sonnenberg JL, Williams-Young D, Ding F, Lipparini F, Egidi F, Goings J, Peng B, Petrone A, Henderson T, Ranasinghe D, Ranasinghe, Zakrzewski VG., Gao J, Rega N, Zheng G, Liang W, Hada M, Ehara M, Toyota K, Fukuda R, Hasegawa J, Ishida M, Nakajima T, Honda Y, Kitao O, Nakai H, Vreven T, Throssell K, Montgomery JA, Jr, Peralta JE, Ogliaro F, Bearpark MJ, JJ, Brothers EN, Kudin KN, Staroverov VN, Keith TA, Kobayashi R, Normand J, Raghavachari K, Rendell AP, Burant JC, Iyengar SS, Tomasi J, Cossi M, Millam JM, Klene M, Adamo C,

Cammi R, Ochterski JW, Martin RL, Morokuma K, Farkas O, Foresman JB, and Fox DJ, Gaussian, Inc., Wallingford CT, (2016).

(10) Weigend, F. & Ahlrichs, R. Balanced basis sets of split valence, triple zeta valence and quadruple zeta valence quality for H to Rn: design and assessment of accuracy. *Physical Chemistry Chemical Physics* **7**, 3297-3305 (2005).

(11) Weigend, F. Accurate coulomb-fitting basis sets for H to Rn. *Physical Chemistry Chemical Physics* **8**, 1057-1065 (2006).

(12) Glendening, E. D., Landis, C. R. & Weinhold, F. NBO 7.0: new vistas in localized and delocalized chemical bonding theory. *J. Comput. Chem.* **40**, 2234-2241 (2019).

(13) Lu T, Multiwfn 3.5 - A Multifunctional Wavefunction Analyzer, School of Chemical and Biological Engineering, University of Science and Technology, Beijing (2018).

(14) Lu T, Chen F. Multiwfn: A multifunctional wavefunction analyzer. *J Comput Chem* **33**, 580-592 (2012).

(15) Humphrey W, Dalke A, Schulten K. VMD: Visual molecular dynamics. *J Mol Graph* **14**, 33-38 (1996).

(16) Legault CY. CYLview, 1.0b, Université de Sherbrooke, Sherbrooke (Québec) Canada, http://www.cylview.org (2009).
